# Artificial intelligence-powered liquid biopsy in cancer: a paradigm shift in cancer detection and personalized care

**DOI:** 10.1186/s12935-026-04263-w

**Published:** 2026-04-02

**Authors:** Sherihan G. AbdelHamid, Esraa M. Halawa, Eman M. Ibrahim, Mahmoud ElHefnawi

**Affiliations:** 1 Department of Biochemistry and Molecular Biology, Faculty of Pharmacy, Ain Shams University, Cairo, 11566 Egypt; 2 Department of Botany and Microbiology, Faculty of Science, Cairo University, Giza, 12613 Egypt; 3 Higher institute for Optics Technology, Sheraton, Cairo, Egypt; 4 Biomedical Informatics and Chemoinformatics Group, Informatics and Systems Department, National Research Centre, Tahrir Street, Cairo, 12311 Egypt

**Keywords:** Artificial intelligence, Machine learning, Liquid biopsy, Breast cancer, Lung cancer, Colorectal cancer, Hepatocellular carcinoma

## Abstract

Liquid biopsy has evolved as a transformative strategy revolutionizing the oncology field. It encompasses the detection of circulating biomarkers derived from tumors including circulating tumor DNA, extracellular vesicles, tumor educated platelets, circulating tumor cells, and circulating RNAs within body fluids. Nonetheless, its application in the clinical settings continues to be constrained by issues related to limited sensitivity, specificity, and lack of standardization. This review uniquely examines the convergence of artificial intelligence (AI) and machine learning (ML) algorithms with liquid biopsy to overcome these barriers and advance precision oncology. Focusing on four major malignancies; breast, lung, colorectal cancers, and hepatocellular carcinoma; we critically evaluate how AI-powered liquid biopsy improves early detection of cancer, prognosis prediction, and monitoring treatment response, while also forecasting recurrence and enabling patient stratification. We further highlight emerging algorithmic innovations, translational challenges, and ethical considerations, emphasizing the urgent need for harmonized validation frameworks to ensure reproducibility and clinical adoption. By leveraging AI-driven molecular insights, liquid biopsy can transition from a research concept to a routine clinical assay, enabling individualized therapeutic strategies, improving long-term survival, and ultimately transforming cancer care into a more predictive, personalized, and patient-centered paradigm.

**Graphical Abstract.:**

**Figure:**
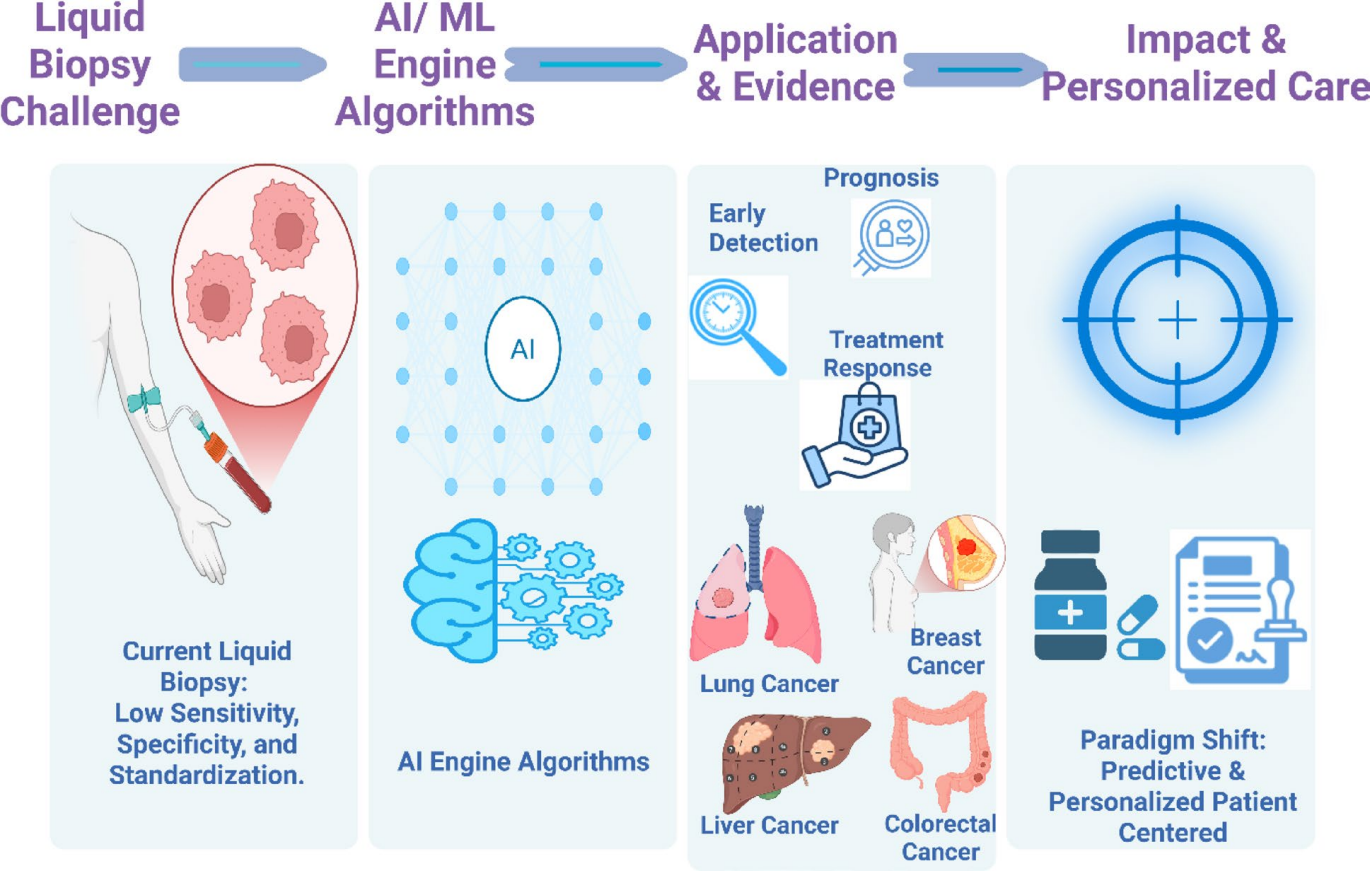
Created in BioRender. Galal, S. (2026) https://BioRender.com/1zu7u3r

## Introduction

Cancer poses substantial health burden worldwide, with forecasted increase in incident cases to 29 million by 2040 [1]. In order to address the global rise in cancer mortality rates, synchronized endeavors directed to early diagnosis, predicting prognosis and personalized treatment strategies are urgently needed to improve patients’ outcomes and decrease the immense strain on healthcare systems worldwide. Current research in the field of clinical oncology is increasingly centered on understanding the intricate biological mechanisms underlying cancer growth, with the aim of unravelling the molecular foundations of cancer development [2].

Liquid biopsy has evolved as a transformative strategy revolutionizing the oncology field [3]. It encompasses the detection of circulating biomarkers derived from tumors including circulating tumor cells (CTCs), circulating tumor DNA (ctDNA), extracellular vesicles (EVs), tumor-educated platelets (TEPs), and circulating RNAs within body fluids [4]. Liquid biopsy could ultimately uncover the genetic and molecular alterations that occur early in tumorigenesis and provide circulating molecular portraits of the tumor. It represents a burgeoning field in precision oncology improving early detection, predicting prognosis, and monitoring treatment response in different malignancies [4].

## The foundation: understanding liquid biopsy and cancer prognosis

### Liquid biopsy: a minimally invasive lens into cancer

Liquid biopsy has particularly evolved as a groundbreaking strategy, offering a minimally invasive lens of monitoring the status of cancer [5]. Unlike conventional tissue biopsy, which requires surgical excision of tumor material [6], liquid biopsy relies on a simple blood draw to detect tumor-derived material, such as intact cancer cells and fragmented tumor DNA. This approach provides a safer and more accessible option, particularly in cases where tumor sampling through surgery is challenging [7]. Although tissue biopsy continues to represent the diagnostic “gold standard,” liquid biopsy is increasingly recognized as a powerful complementary tool that captures dynamic insights into tumor biology and evolution [8].

**Fig. 1:**
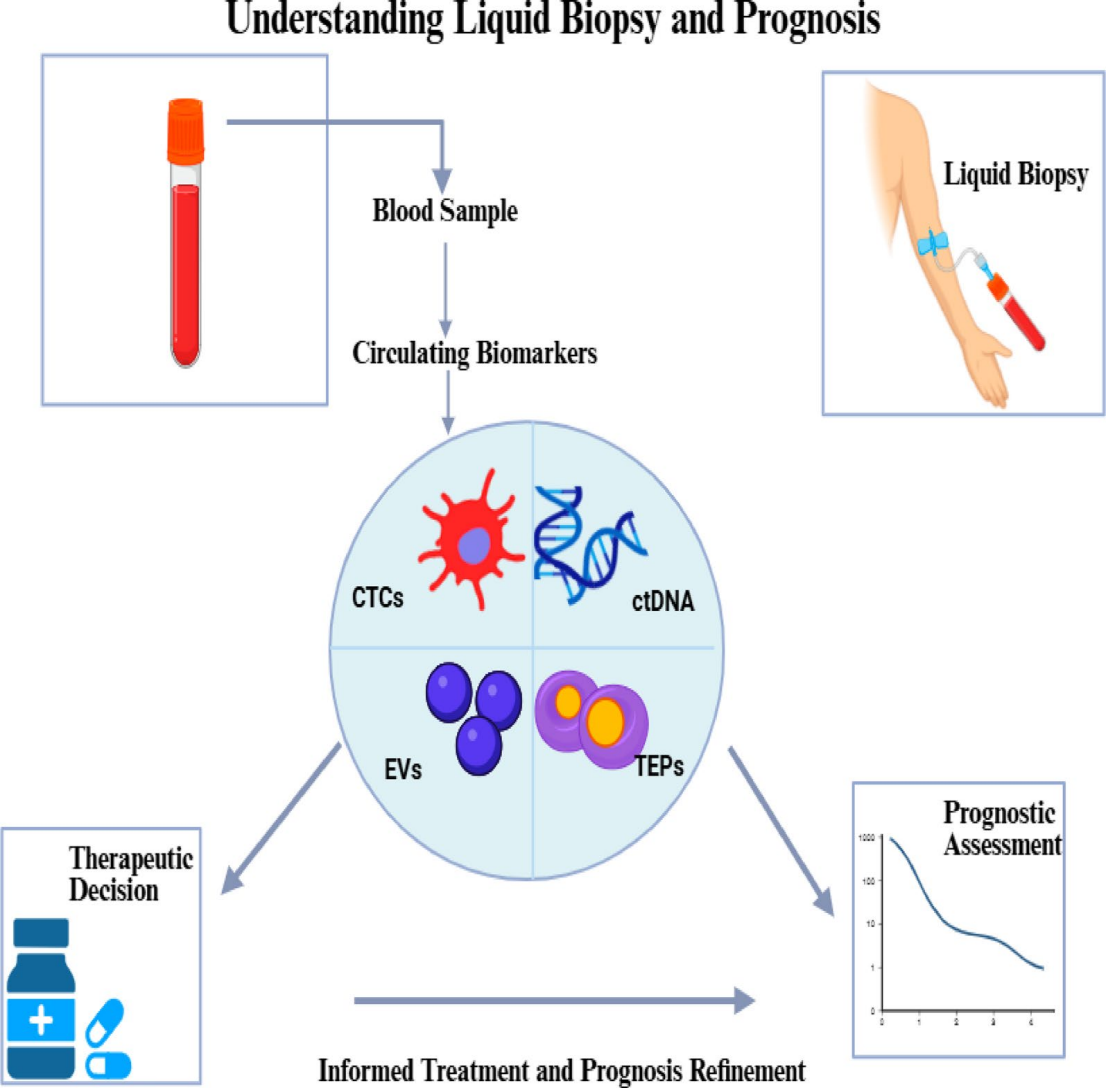
The foundation: understanding liquid biopsy and cancer prognosis

CTCs: Circulating tumor cells; ctDNA: Circulating tumor DNA; EVs: Extracellular vesicles; TEPs: Tumor-educated platelets. Created in BioRender. Galal, S. (2026) https://BioRender.com/3afm1be

Recent advances in identification of different liquid biopsy biomarkers have greatly enhanced its potential application in the clinical settings, as they portray the molecular landscape of the tumor. By interrogating tumor-derived analytes circulating in body fluids (most commonly blood), liquid biopsy enables the identification of several key biomarkers relevant to cancer prognosis (Fig. 1):

1. **Circulating Tumor Cells**: CTCs are intact cancer cells detached from the tumor and enter the bloodstream [9]. They reflect phenotypic as well as molecular information about the tumor and its potential for metastasis. However, their scarcity, frequently fewer than 50 cells in a volume of 7.5 mL blood sample, poses significant technical challenges for their detection and enrichment [10].
2. **Circulating Tumor DNA**: ctDNA consists of DNA fragments shed from apoptotic or necrotic cancer cells into circulation [11]. ctDNA represents a subset of cell-free DNA (cfDNA) that encompasses fragments circulating freely in the bloodstream, originating from various sources including normal cell turnover, primarily hematopoietic cells, apoptosis, necrosis, and active cellular secretion [12]. Hence, in cancer patients, ctDNA typically represents only a small fraction (0.01% to > 10%) of total cfDNA, with the remainder being “background” cfDNA from normal cells. ctDNA offers a wealth of tumor-specific alterations including somatic mutations, epigenetic signatures such as DNA methylation profiles, and chromosomal rearrangements [13].
3. **Exosomes and Extracellular Vesicles**: These nanoscale, lipid-enclosed vesicles carry nucleic acids and proteins [14]. Tumor-derived exosomes have gained increasing attention as prognostic and diagnostic biomarkers, as they mediate intercellular signaling and mirror the molecular landscape of their tumor of origin [15].
4. **Tumor-Educated Platelets**: These are platelets that have interacted with tumors and have undergone molecular reprogramming and can be profiled for their RNA cargo, providing another dynamic layer of information about the tumor’s biology [16].

Together, these circulating biomarkers not only signal the presence of malignancy but also supply valuable genetic and molecular information that can inform therapeutic decisions and refine prognostic assessment [17]. A structured comparison of these analytes underscoring the distinct types of information they provide, which in turn influences the analytical approach and the clinical questions they can address, is depicted in (Table 1**)**.

**Table 1:** Liquid biopsy biomarkers: applications and technical challenges

Analyte	Nature of data	Prognostic application	Technical challenges
**Circulating Tumor Cells (CTCs)**	Whole cells with intact genomic, transcriptomic, and proteomic profiles [18].	Association with aggressive disease and poor prognosis; tracking metastatic potential; real-time monitoring of therapeutic response [18].	Extreme rarity in the bloodstream (< 50 CTCs in 7.5 ml of blood); technical difficulty in isolation and purification [18].
**Circulating Tumor DNA (ctDNA)**	Fragments of DNA, shed from tumor carrying genetic variants [19].	Predicting recurrence via minimal residual disease (MRD) detection; informing targeted therapy selection; assessing drug resistance [19].	Low concentration in early-stage cancers; potential for false negatives due to limited shedding; need for timely analysis due to short half-life [19].
**Exosomes/Extracellular Vesicles (EVs)**	Lipid-bilayer spheres containing nucleic acids and proteins [20].	Emerging biomarkers for cancer diagnosis and prognosis; potential to reveal data about the tumor microenvironment [20].	Lack of standardized isolation protocols; low throughput for some isolation methods [20].
**Tumor-Educated Platelets (TEPs)**	Platelets that have taken up tumor-derived materials, including RNA [21].	Used for cancer classification and early detection based on RNA sequencing data [21].	Data interpretation requires sophisticated bioinformatics and machine learning models [21].

However, each of these biomarkers possesses inherent strengths and limitations, ranging from the scarcity of CTCs to the fragmented nature of ctDNA. The true potential of liquid biopsy lies in the joint analysis of these disparate signals, a task that introduces significant multidimensional complexity. Because these analytes often yield discordant or subtle molecular patterns, their integration calls for powerful computational tools to interpret the high-dimensional data, filter biological noise, and extract actionable prognostic signatures [22, 23].

### The prognostic imperative: liquid biopsy vs. tissue biopsy

In clinical oncology, the significance of liquid biopsy extends beyond diagnosis, it lies in its capacity to generate prognostic insights, helping to anticipate the likely trajectory of a patient’s disease [24]. For instance, CellSearch^®^ CTC is a Food and Drug Administration (FDA) approved test which stratifies outcomes in patients with metastatic breast, prostate, or colorectal cancer (CRC), where lower CTC counts are associated with improved outcome [25]. The non-invasive and repeatable nature of liquid biopsy allows longitudinal follow-up, offering clinicians a real-time view of disease evolution and response to therapy [24].

Rather than being viewed as competing modalities, tissue and liquid biopsies should be considered complementary. Tissue biopsy provides a detailed histopathological and molecular characterization of a tumor at a single time point [26]. However, this snapshot is limited by tumor heterogeneity, as a single tissue sample may fail to capture the diversity of subclones within the primary lesion or its metastases. In addition, tissue collection can be invasive, carries procedural risks, and may be unfeasible for tumors in anatomically inaccessible regions [27].

**Fig. 2:**
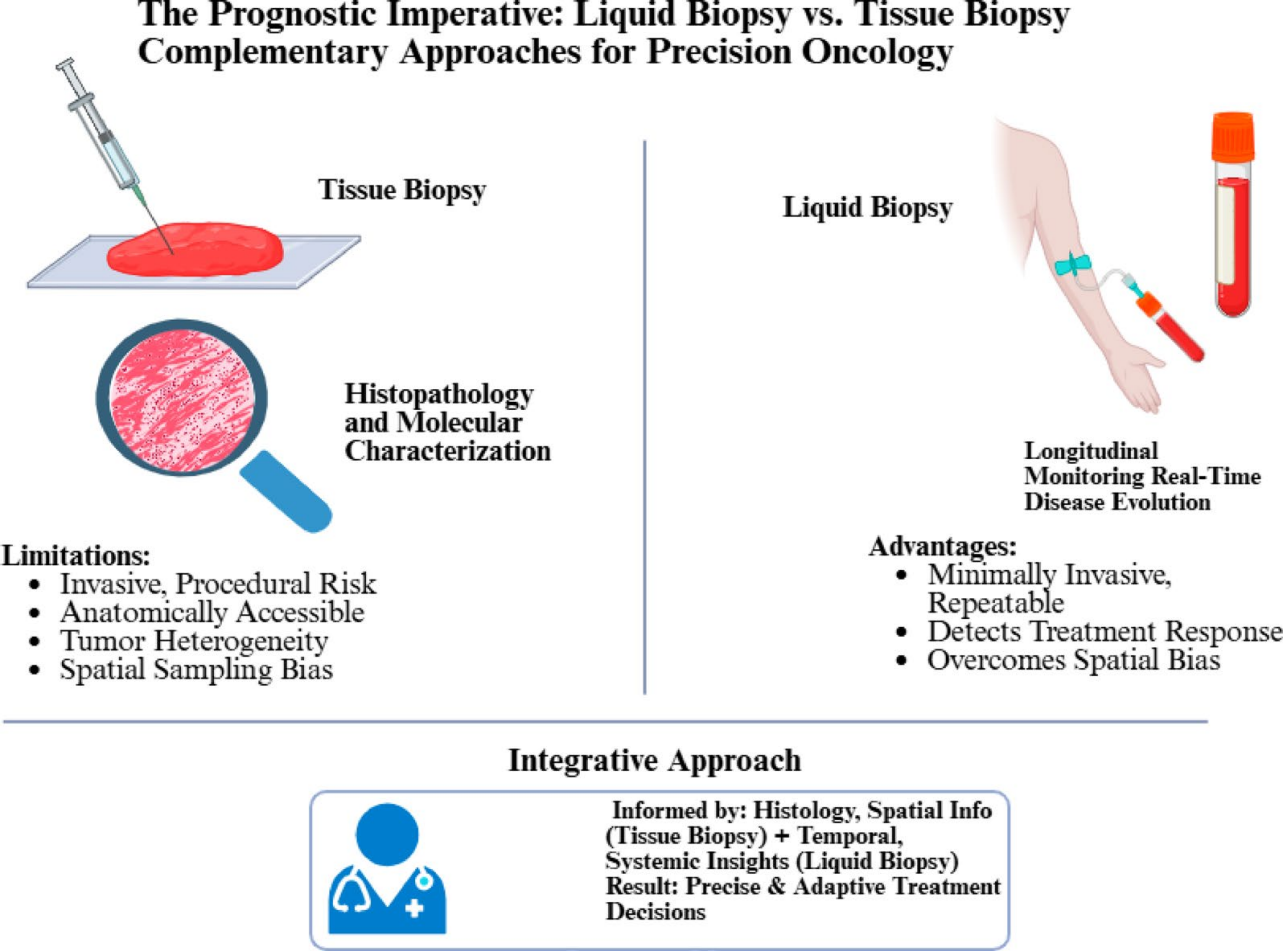
The Prognostic Imperative: Liquid Biopsy vs. Tissue Biopsy. CTCs: Circulating tumor cells; ctDNA: Circulating tumor DNA; TEPs: Tumor-educated platelets. Created in BioRender. Galal, S. (2026) https://BioRender.com/2gnn5kt

Liquid biopsy, by contrast, provides a dynamic overview of tumor behavior. Its minimally invasive nature facilitates serial sampling, allowing the detection of treatment response, clonal evolution, and emerging resistance mechanisms. Owing to its short half-life, ctDNA is a particularly sensitive biomarker for reflecting changes in tumor burden in near real-time. Importantly, because circulating analytes originate from multiple tumor sites, liquid biopsy can help overcome the spatial sampling bias inherent to tissue biopsies [5]. Nonetheless, limitations remain. Liquid biopsy cannot provide architectural or histological details, and its sensitivity can be inconsistent, especially in cancers that shed little material into circulation or at early disease stages [28]. Consequently, the optimal approach is an integrative one leveraging the histological and spatial information obtained from tissue biopsy alongside the temporal and systemic insights of liquid biopsy **(**Fig. 2**)**. Together, these complementary tools empower clinicians to make more precise and adaptive treatment decisions [29].

## The computational engine: machine learning for complex biological data

### Why machine learning is critical for liquid biopsy

Liquid biopsy, a non-invasive methodology that investigates key circulating biomarkers, provides a comprehensive, real-time molecular overview of tumor evolution and facilitates continuous monitoring of treatment response, which is a major advantage over static tissue sampling. However, the efficacy of liquid biopsy faces technical hurdles, including reduced sensitivity for early-stage disease and potential decreased detectability resulting from hepatic filtration of biomarkers [30].

In addition, one of the biggest challenges in liquid biopsy is the low signal-to-noise ratio, that is distinguishing tumor-generated ctDNA from the large background noise of cfDNA derived from hematopoietic cells and other non-malignant tissues [31]. Artificial intelligence (AI) models are indispensable to accomplish this discrimination through multi-layered analytical strategies exploiting distinct molecular features that distinguish ctDNA from normal cfDNA. This include somatic mutation detection, differential DNA methylation patterns, and copy number alterations [32]. In addition, advanced AI algorithms harness high-dimensional fragmentomic features to boost cancer detection precision, analyzing fragment size profiles and end motifs [33]. Because of abnormal nuclease activity in the tumor microenvironment, ctDNA usually shows fragmentation shortening (approximately143–145 bp) and unique 4-mer end-motif frequencies, whereas cfDNA preserves a classic ~ 166 bp nucleosomal footprint. AI model efficiently overcomes the intrinsic noise of systemic physiological turnover by performing single-molecule inference using a Transformer-based architecture and dynamically weighting these fragmentomic features to enable in silico enrichment of the tumor signal [3]. Deep learning (DL) architectures, particularly convolutional neural networks (CNNs) and recurrent neural network (RNNs), excel at automatic feature extraction from raw sequencing data by learning hierarchical representations that capture patterns across multiple scales [34]. Ensemble methods (like Random Forests (RFs) and Gradient Boosting) combine weak learners to amplify subtle ctDNA signals imperceptible to single classifiers, improving sensitivity for low tumor fraction samples. Denoising autoencoders learn to reconstruct clean signals from noisy inputs by training on high-quality paired datasets, while transfer learning leverages large multi-institutional datasets to develop generalizable cancer signatures robust across diverse populations and technical platforms [35].

Liquid biopsy technologies also produce complex, heterogenous multi-layered datasets, commonly referred to as multi-omics spanning genomic, transcriptomic, proteomic, and epigenomic information [36, 37]. These datasets are inherently high-dimensional, with far more features (biomarkers) than samples, and are often complicated by biological variability and noise. Traditional statistical and diagnostic approaches, constrained by human interpretive capacity, are insufficient for uncovering the non-linear interactions that underpin tumor biology [38]. AI has produced a paradigm revolution in scientific research via enabling data analysis and discovery. AI, particularly machine learning (ML) techniques, presents an evolutionary solution by extracting clinically relevant patterns from such complexity. ML algorithms autonomously identify hidden associations in large datasets without requiring explicit preprogramming [39]. This is particularly valuable for liquid biopsy, where reliance on a single biomarker often fails to portray the entire scope of tumor heterogeneity and interpatient variability. By integrating diverse circulating biomarkers, ML models can identify composite predictive signatures that are beyond human discernment [40].

**Fig. 3:**
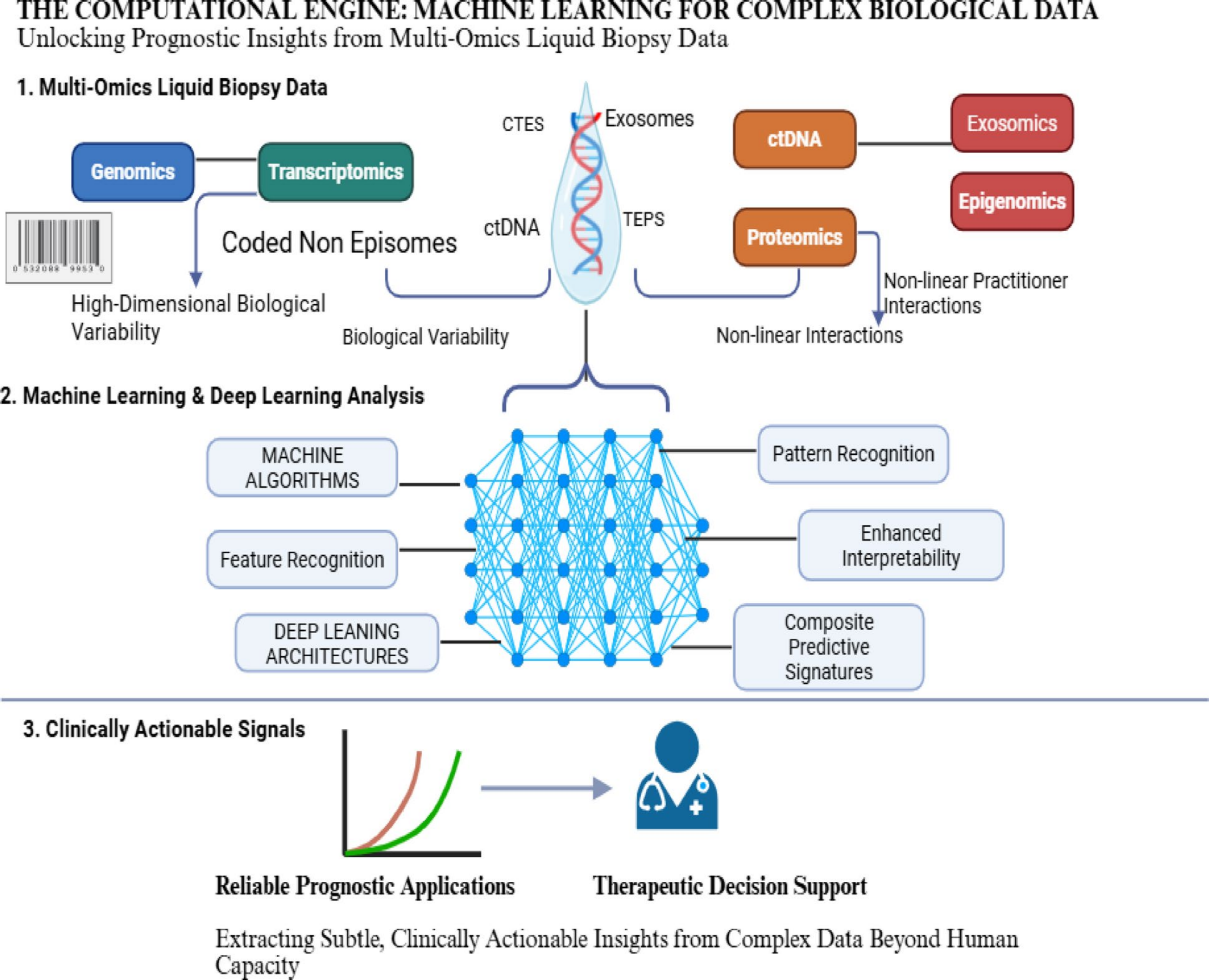
The Computational Engine: Machine Learning for Complex Biological Data. Created in BioRender. Galal, S. (2026) https://BioRender.com/i98c1iw

Ultimately, the reliance on ML is a direct response to the scale and intricacy of liquid biopsy data. Only through advanced computational models can subtle, clinically actionable signals be distinguished from background variability, enabling reliable prognostic applications [41]. Accordingly, the integration of ML for complex biological data could ultimately unlock prognostic insights from multi-omics liquid biopsy data (Fig. 3).

### A taxonomy of machine learning and deep learning models

A broad spectrum of ML and deep learning (DL) approaches has been integrated to analyze liquid biopsy datasets to support cancer diagnosis, prognosis, and therapeutic stratification. Model selection typically relies on the type of data and the particular analytical goal—classification (e.g., cancer vs. non-cancer) or regression (e.g., survival prediction) [40].

#### Traditional machine learning models

(A). * Support Vector Machines (SVMs)*: High Efficiency for high-dimensional data, SVMs separate diagnostic groups by maximizing the margin between classes in feature space. They have demonstrated success in identifying protein biomarkers for classification, particularly in smaller datasets requiring robust accuracy [42].
(B). * Decision Trees (DTs)*,* K-Nearest Neighbor (KNN)*,* and Naïve Bayes*: Widely used in medical diagnostics, these models have shown strong utility in handling structured clinical and molecular data. Ensemble methods such as ** RFs)**—which combine multiple DTs, have been applied alongside SVMs to improve biomarker discovery and manage high-dimensional datasets [43].

#### Deep learning models

(A). * Convolutional Neural Networks (CNNs)*: Conventionally employed in image analysis [44], CNNs have been implemented for analyzing liquid biopsy by transforming gene expression data (e.g., platelet RNA) into heatmap-like inputs. This approach leverages CNNs’ strength in pattern recognition, achieving classification accuracies of up to 90.5% in certain cancer types [45].
(B). * Fully Connected Neural Networks (NNs)*: Multi-layered artificial neural networks capable of integrating heterogeneous data sources. These models excel in extracting higher-order, abstract relationships from complex biological data and have demonstrated strong performance in cancer prognosis, often surpassing classical models such as Cox proportional hazards (Cox-PH) [46, 47].

The application of these specific models demonstrates the nuanced approach required for different types of liquid biopsy data. A model’s suitability depends on the data’s structure. For example, a CNN is well-suited for image-based data, such as a heatmap generated from TEP RNA, whereas an SVM is an effective tool for classifying high-dimensional data like protein or gene expression profiles [48]. This tailored application of ML algorithms is a critical step in deriving meaningful insights from the varied and complex data streams produced by liquid biopsies (Table 2).

**Table 2:** Specific applications of machine learning models in liquid biopsy

Machine learning model	Core function	Specific application in liquid biopsy
**Support Vector Machine (SVM)**	Supervised classification and regression.	Identifying protein biomarkers to distinguish between cancer sites; handling high-dimensional omics data; particularly useful for small datasets with high accuracy demands [49].
**Decision Tree (DT)/Random Forest (RF)**	Categorization and regression using a divide-and-conquer approach.	Disease diagnosis and biomarker identification; used in conjunction with other algorithms to select diagnostic markers from high-dimensional data [50].
**Convolutional Neural Network (CNN)**	Pattern recognition and feature extraction from grid-like data.	Analyzing heatmap images generated from blood platelet RNA to classify cancer types; used in AI-based liquid biopsy approaches for brain cancer detection [51].
**Fully Connected Neural Network (NN)**	Mapping complex relationships between input and output data.	Cancer prognosis prediction with multi-omics data; can accept various data formats and requires less feature engineering [52, 53]

Building on the computational frameworks previously discussed, AI models are now being integrated into clinical workflows to push the limits of early detection and personalized monitoring.

## The synergy in action: clinical applications and evidence in transforming cancer care

**Fig. 4:**
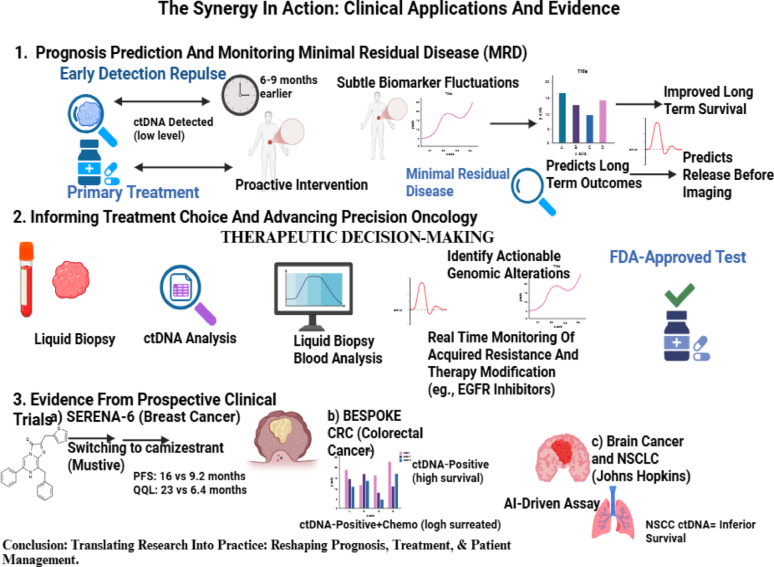
The Synergy in Action: Clinical Applications and Evidence. Created in BioRender. Galal, S. (2026) https://BioRender.com/yftjvw1

Having established the taxonomy of AI models and their specific technical strengths in handling high-dimensional biological data, it is essential to examine how these computational “engines” translate into tangible patient outcomes. This section discusses the clinical impact of the synergistic integration of AI and ML techniques with liquid biopsy in transforming cancer care through improving early detection of cancer, predicting prognosis, and monitoring treatment response, while also forecasting recurrence and enabling patient stratification, focusing on four major malignancies: breast, lung, CRC, and hepatocellular carcinoma (HCC). The clinical applications and evidence of the role AI-powered liquid biopsy in cancer is outlined in Fig. 4.

### Role of AI-powered liquid biopsy in early detection of cancer

Early cancer detection constitutes a major challenge and crucial determinant of survival. Diagnosis of cancer at the earliest stage paves the way for timely and effective management, ultimately improving prognosis and quality of life [54]. Traditional screening/diagnostic modalities like tissue biopsy and different imaging techniques are invasive and expensive, in addition to limited sensitivity and specificity. Liquid biopsy is a promising noninvasive method for detection of cancer through the analysis of circulating tumor derived biomarkers in body fluids [4]. In early stages of cancer, the amount of these biomarkers may be very low to allow their accurate identification by traditional statistical and computational methods due to low signal-to-noise ratio, in addition to data complexity and detection variability [4]. DL networks with strong pattern-recognition capabilities can learn and amplify faint, multidimensional cancer signals, thereby pushing the sensitivity of liquid biopsy to new limits [55].

The incorporation of multi-omics data generated from liquid biopsy could be challenging due to the complexity of datasets, biological variability, and data noise. The integration of AI, particularly ML, with liquid biopsy can greatly overcome those challenges [56]. By enabling multi-omics integration, biomarker discovery and predictive modelling, ML models could process complex datasets allowing a more comprehensive approach to the diagnosis of different malignancies.

Several ML methods have been developed for early cancer diagnosis utilizing liquid biopsy samples for the analysis of cfDNA mutations together with protein biomarkers [57]. An example is CancerSEEK model, that involves the use of cfDNA/ctDNA mutation data and protein biomarker concentrations to discriminate cancer patients from healthy individuals with 99% specificity [58]. The test was validated with 1005 cancer patients with 8 types of nonmetastatic cancers (esophagus, breast, ovarian, stomach, pancreas, liver, CRC, lung cancers) and 812 healthy subjects [58]. Based on the same dataset, Wong et al. developed CancerA1DE system that relied on Aggregating One-Dependence Estimators (A1DE) which could detect cancer cases with 96.64% accuracy [59]. In addition, Rahman et al. developed CancerEMC system that achieved 74.12% accuracy for localized multiple cancer detection and 99.17% accuracy for binary cancer classification [60].

Cristiano et al. developed an approach named “DNA evaluation of fragments for early interception” (DELFI) for the screening, early detection and monitoring of human cancer through the analysis of the fragmentation profiles of 236 patients with breast, ovarian, pancreatic, gastric, colorectal, bile duct, or lung cancer and 245 healthy subjects. The integration of ML model to analyze these genome-wide fragmentation features could detect the seven cancer types at 98% specificity with detection sensitivities from 57% to 99% [33].

DEcancer is another ML pipeline for detection of cancer that analyzes clinical data obtained from blood tests [61]. It demonstrated increased sensitivity for the detection of stage 1 cancers across different types to 90% [60]. Eleddkawy et al. proposed a system for cancer diagnosis utilizing protein biomarker concentrations and mutations in plasma cfDNA/ctDNA [57]. The system enables early detection and classification of breast, CRC, ovarian, upper gastrointestinal (GI), liver, pancreas, and lung cancers. It utilizes correlation coefficient to detect features that are correlated within the dataset, followed by the assessment of the relevancy of each pair to the target variable. The system utilizes XGBoost feature to sort out the 10 most important features, enhancing the model interpretability in a significant way [57].

ML models can also analyze complex data from multiple sources including DNA methylation, messenger RNA (mRNAs), long non-coding RNAs (lncRNAs) and microRNAs (miRNAs) to classify various tumors (ovarian, breast, and brain cancers) into subtypes providing insights for improved patient management [62, 63]. In addition, a kernel-based ML algorithm could evaluate the predictive value of genomic, epigenomic and transcriptomic data of various cancers [64]. ML approaches have also been implemented to investigate large-scale epigenomic data to pinpoint patterns pertaining to particular cancer, potentially offering candidate biomarkers for precise and early diagnosis [65]. The integration of ML algorithms with RNAseq data enables the detection of promising genetic signatures and biomarkers that are crucial for deciphering tumor biology and evaluating patient outcomes [13]. Also, the integration of AI-assisted analysis of metagenome data can improve the identification of early molecular changes, potentially enhancing detection of cancer at incipient stage [66].

CTCs have been recognized as crucial biomarkers in clinical oncology research. Owing to their heterogeneity, the integration of ML approaches in the analysis of CTCs including RFs, SVM, and naive Bayes classifiers could overcome the challenges and enhance the accuracy of CTCs detection [67]. A validation study that utilized trajectory-based ML classification of CTCs in microfluidics allowed the differentiation of different CTCs phenotypes and hence enabled early detection of cancer [68]. Also, Park et al. developed a hybrid algorithm, CNN-SVM, for the categorization of CTC clusters based on morphological characteristics with a sensitivity and specificity exceeding 90%, suggesting its role as a promising approach for cancer detection and predicting prognosis [69].

Multi-cancer early detection (MCED) tools are liquid biopsy-based tests that can assess different cancer hallmarks via measuring aberrant DNA methylation patterns, DNA mutations, cfDNA fragmentomics, and epigenetic biomarkers, enabling simultaneous screening for multiple cancers [70]. GRAIL’s Galleri test is an MCED tool powered by ML that analyze cfDNA methylation. It utilizes DL together with AI-driven classifiers. In a clinical validation sub study, the MCED tool showed enhanced accuracy in identifying the origin of the cancer signal and identified signals relevant to more than 50 cancer types [71, 72]. Another sub study on cfDNA-based MCED showed that circulating tumor allele fraction can more closely reflect tumor biology and was a significant predictor of classifier performance, while whole genome methylation feature was the best to predict cancer signal origin [73].

Multimodality AI models can integrate complex genetic, epigenetic, proteomic and transcriptomic data from liquid biopsy together with radiological and pathological information to provide a clear image to differentiate benign and malignant lesions [55]. For instance, the combination of multimodal analysis of cfDNA methylome, fragmentome and copy number variations with ensemble ML algorithms in plasma samples of 780 cancer patients (Breast, CRC, gastric, esophagus, pancreas, liver and lung cancers) and 497 healthy controls could accurately detect cancer with AUC of 0.966 at 73% sensitivity and 99% specificity for early diagnosis of cancer with accurate localization of the origin of cancer [74]. Another multimodal assay named (Screening for the Presence of Tumor by Methylation and Size (SPOT-MAS) was utilized in a single workflow to analyze fragmentomics end motifs data, methylomics, copy number variations obtained by cfDNA through utilizing targeted and shallow genome-wide sequencing in 1550 healthy controls and 738 patients suffering from non-metastatic CRC, lung, liver, breast, and gastric cancers. The assay was able to detect cancer at 72.4% sensitivity and 97.0% specificity [75]. Natural language processing (NLP) could also extract diagnostic information from electronic medical records, further helping in assessing cancer risk and need for further screening [76, 77].

The current applications of AI cover several areas of cancer research including genomics, proteomics, metabolomics, transcriptomics, immunomics and microbiomics, in addition to pathomics and radiomics. In the current section, we will unravel the role of AI-powered liquid biopsy in early detection of breast cancer, lung cancer, CRC, and HCC.

#### AI-powered liquid biopsy in early detection of breast cancer

Breast cancer is the most frequently diagnosed malignancy and is also the leading cause of cancer-related mortality among females globally [1]. Although mammography has been recognized as the gold standard for breast cancer screening, yet several limitations exist including low sensitivity and probability of false positive results [78]. Accordingly, there is a crucial need for specific and accurate biomarkers to allow early breast cancer detection. Liquid biopsy has demonstrated its potential as non-invasive technique that can identify small amounts of tumor-derived biomarkers allowing early detection [79]. Integration of advanced AI tools with liquid biopsy enables the detection of breast cancer at the earliest stage before clinical symptoms become evident, allowing the implementation of timely and targeted treatment strategies.

Aberrant DNA methylation plays a significant role in breast carcinogenesis that often arises in early stages of tumor development, rendering them potential biomarkers for early diagnosis [79]. The integration of ML with both single-omics and multi-omics data could identify potential DNA methylation-based biomarkers which have been detected in precancerous or incipient stages of breast cancer, hence improving the sensitivity of early detection [79]. Gomes et al.. proposed a DL workflow incorporating feature engineering techniques (imputation and data balancing tools), to predict and detect methylation-based biomarkers that could diagnose breast cancer with high accuracy [80].

Wang et al. utilized the RF algorithm to construct a methylation-based approach to classify breast cancer and predict breast tumor invasiveness [81]. Based on DNA methylation profile, a deep embedded refined clustering system was employed to classify breast cancer and hence aid in diagnosis [82]. Moreover, the amalgamation of methylation data with molecular signatures as proteomics and transcriptomics using ML techniques could improve the accuracy in breast cancer diagnosis [41]. For example, Xu et al. proposed a supervised ML approach utilizing multi-omics feature selection of DNA methylation, copy number variations and transcriptomic data together with model regularization for the identification of 10-gene signature panel for diagnosing breast cancer and stratifying patients with high accuracy [83].

An innovative DL model was developed utilizing Bayesian optimization for the integration of DNA methylation, mRNAs, miRNAs, gene mutations and MRI data with breast cancer molecular characteristics to improve the accuracy of triple negative breast cancer (TNBC) classification and prediction of prognosis [84]. SPOT-MAS, a multimodal liquid biopsy test was developed and applied for the analysis of cfDNA methylation with copy number alterations and fragmentomics (end motif) that could detect breast cancer at early stage with very high accuracy (AUC of 0.91, 96% specificity and 65% sensitivity) [85].

In another study, the combined investigation of DNA methylation profile, genetic, and gene expression data demonstrated that 38 DNA methylation biomarkers could impact breast cancer risk via the regulation of expression of 21 genes [86]. A systematic review demonstrated that conventional ML methods could detect breast cancer with 90% accuracy, while RNN outperformed them achieving 98.58% acuracy [87].

Moreover, another study integrated supervised ML algorithms with detection of CTCs and tumor microenvironment cells in blood samples of 534 patients diagnosed with breast cancer and healthy controls together with immunofluorescence and cellular features data. This approach could achieve high accuracy in early detection of breast cancer approaching 98.8% [88].

The combination of AI with EVs analysis in liquid biopsy shows a significant potential in breast cancer research, enabling non-invasive and more precise diagnostic options which can help in early breast cancer detection [89]. Hoshino et al. investigated the proteomic profile of EVs and particles in 426 cancer patients including breast cancer from body fluids as blood and tissue explants [90]. ML categorization of EVs cargo derived from plasma achieved 95% sensitivity and 90% specificity in detection of cancer. In addition, they could define a panel of proteins derived from plasma that is specific to each tumor type. These EVs protein signatures pertinent to specific cancer types could be used as a promising liquid biopsy approach in the diagnosis and management of cancer patients [90].

In addition, Zhang et al. pioneered a methodology combining CNN with Total Internal Reflection Fluorescence (TIRF) imaging that could detect multiple miRNAs simultaneously and accurately at the level of a single EV. Their evaluation confirmed the heterogeneous nature of EV miRNA expression, noting that the triple-positive EV subpopulation was the primary differentiator between EVs extracted from different cancer cell types and normal plasma. The CNN-based approach achieved a high classification accuracy (above 95%) and demonstrated perfect accuracy (100%) in a small clinical cohort of 20 patients, including breast cancer, alongside lung, cervical, and colon cancer cases, when compared to healthy controls [91].

Although MCED blood tests are not yet approved for population screening, prospective studies are ongoing to elucidate multi-omics plasma signature that could detect asymptomatic cancers and guide targeted imaging in breast cancer. In addition, integration of plasma metabolomics and proteomics could identify amino-acid and redox-pathway signatures distinguishing patients with breast cancer or benign disease with AUCs of 0.79–0.88 [92].

Breast cancer patients could also be stratified into high and low risk groups through the integration of proteomics and metabolomics data. Choi et al. developed moBRCA-net, a breast cancer subtype classification framework based on multi-omics attention neural networks, where autoencoders and multiple-kernel frameworks were employed for subtyping [93]. In addition, Huang et al. designed the SALMON method that incorporated traditional biomarkers with multi-omics data through eigengene matrices of co-expression networks in order to distinguish candidate genes and cytobands in breast cancer [94]. A recently published integrative bioinformatic study revealed 12 hub proteins as candidate biomarkers for breast cancer, providing a robust computational basis for future translational research [95].

The ability of AI algorithms to review a large volume of medical data, including imaging data [96], histopathological data [97], together with liquid biopsy data and genetic information, could help in the prediction and early detection of breast cancer [98].

#### AI-powered liquid biopsy in early detection of Lung Cancer

Lung cancer ranks first as the top cause of cancer-related fatalities worldwide, with a five-year survival rate of approximately 20%, leading to the highest death rates among both men and women [1]. Most cases are identified at an advanced stage, which contributes to poor prognosis [99]. Liquid biopsy could enhance lung cancer screening/diagnostic precision by identifying genetic mutations at an earlier stage, before cancer can be detected by imaging techniques like low dose computed tomography (LDCT) [100]. The integration of AI and liquid biopsy has deepened the understanding of complex data, potentially unravelling the molecular mechanisms underlying lung cancer [101].

Integrating AI models can offer a thorough insight into the genetic framework of lung cancer by examining extensive genomic datasets, which facilitates the detection of particular genetic mutations such as epidermal growth factor receptor (EGFR) and KRAS mutations [102–104]. AI algorithms can analyze complex patterns in ctDNA data, potentially identifying cancer-specific signatures before traditional diagnostic methods can detect the disease [75]. Moreover, ML models that utilize serum RNA levels can forecast the onset of lung cancer several years prior to its clinical manifestation. This was demonstrated in a large-scale validation study performed in 1115 high-risk individuals enrolled in a lung cancer screening program. The miRNA test could predict lung cancer with overall 77.8% sensitivity, and 74.8% specificity [105].

AI is a transformative tool in early lung cancer screening [32]. Commercial AI-driven assays, exemplified by the Galleri test (Grail), are engineered to identify lung cancer signals years prior to the manifestation of clinical symptoms or initial radiological visibility [106]. Another example of AI-driven tool for lung cancer detection is Lung Cancer Likelihood in Plasma (CLiP) that applies ensemble-based ML to analyze ctDNA in plasma samples of patients diagnosed with lung cancer. This test enhances the reliability of cancer detection by combining classification algorithms, logistic regression, and DTs [107]. CLiP strategically enhances diagnostic specificity by reliably discriminating tumor-derived ctDNA from background noise including clonal hematopoiesis (CH) [108].

The Orion model is multi-task AI model that was able to analyze orphan non-coding RNAs (oncRNAs) from blood samples of 1050 non-small cell lung cancer (NSCLC) patients at various stages, together with age and sex matched- healthy controls, at 87% overall specificity and 94% overall sensitivity [109]. This model utilizes semi-supervised variational autoencoders on oncRNA data to derive a Gaussian distribution, modeling the expression of oncRNAs and annotated small RNAs. It integrates stratification and contrastive learning objectives to enhance label prediction and eliminate any confounding factors [109].

DEcancer is an AI framework that used a liquid biopsy proteomics dataset of a cohort of 61 lung cancer patients and 80 normal individuals. It could detect cancer using a 14–43 protein panel and the performance was similar to 1,000 original proteins [61].

The recent trends in lung cancer screening include the integration of AI not only with biomarkers-based liquid biopsy but also with LDCT [100, 110]. Ye et al. developed a classifier model incorporating AI with liquid biopsy and LDCT data on a training set of 560 individuals having moderate to high risk of developing lung cancer [111]. The model provided diagnostic performance with 89.5% sensitivity and 81.31%, specificity [111].

Recent advances include integrating fragmentomics, which analyzes the size and pattern of circulating DNA fragments [112]. AI algorithms can interpret these complex patterns to discriminate tumor DNA from normal DNA with high precision. This approach shows particular promise in early lung cancer detection and in tracking minimal residual disease (MRD), where traditional ctDNA testing sometimes falls short [113, 114]. A prospective study employing an ML model for measuring tumor-derived cfDNA through genome-wide analyses of cfDNA fragmentation was carried out in 365 individuals at risk for lung cancer and was further validated using a cohort of 46 lung cancer patients and 385 non-cancer individuals. Integration of fragmentomics features, CEA levels, and clinical risk factors, followed by CT imaging, detected 94% of patients at 80% specificity [115].

AI can provide highly precise and effective medical solutions through integrating and leveraging genetic, clinical, and imaging data, significantly enhancing patient outcome and quality of life [101]. A multicenter prospective study conducted by Visser et al. assessed the effectiveness of liquid biopsy decision support algorithms for the detection and stratification of lung cancer. They employed multi-parametric approached to evaluate ctDNA mutations in EGFR, KRAS and BRAF genes as well as eight protein tumor markers; CA125, CA15.3, CEA, CYFRA 21 − 1, SCCA, proGRP, HE4, and NSE; in blood of 1096 patients suspected of having lung cancer [116]. The liquid-biopsy–based decision–support algorithms successfully identified approximately 75% of all lung cancer and NSCLC patients and 50% of small-cell lung cancer patients, underscoring their potential clinical value [116].

#### AI-powered liquid biopsy in early detection of colorectal cancer

CRC represents the third most prevalent cancer globally and the second leading cause of cancer-related deaths [1], with 60% anticipated increase in worldwide incidence and mortality rates by 2030 [117]. Nevertheless, early diagnosis and timely intervention can greatly improve the survival. Current diagnostic modalities including colonoscopy, histopathological examination of tumor biopsy samples, conventional circulating tumor markers like CEA and CA19-9, and imaging techniques have several limitations of either invasiveness or low sensitivity and/or specificity. Accordingly, they have limited clinical utility in the early detection of CRC, when timely intervention can improve patients’ outcome [118]. Liquid biopsy offers great potential in real-time monitoring of the molecular profile of the tumor, enabling diagnosis of CRC at earlier stage [119].

The combination of AI and ML with liquid biopsy empowers the processing of complex data and differentiation of malignant and non-malignant alterations, and thus improving diagnostic accuracy [120]. Wan et al. proposed a ML method for identifying CRC at early stages [121]. This approach could reveal cfDNA signatures that reflect alterations from both cancerous and non-cancerous origin. In this study, whole-genome sequencing was carried out on plasma cfDNA of 546 CRC patients and 271 healthy controls and showed the potential to identify CRC at early stages with 85% sensitivity and 85% specificity [121]. In addition, whole-genome sequencing data from plasma cfDNA has been analyzed using AI techniques to detect CRC at early stage. This method has produced strong sensitivity and specificity as well as good diagnostic metrics, such as an AUC of 0.92, especially in cohorts with earlier stage CRC [122].

Acoustic Separation and Concentration of Exosomes and Nucleotide Detection (ASCENDx) is an AI feature extraction approach designed by Naquin et al. [123].The platform enables the diagnosis of CRC with 100% specificity and 95.8% sensitivity via the identification of circulating exosomal miRNAs extracted from blood samples of CRC patients. It uses a spinning droplet and acoustically driven disc rotation to produce swift separation and achieve adequate concentration of plasma-derived exosomes [123]. An FDA-approved AI-driven blood-based test named “Shield” was developed as a screening test for detection of different cancers and could detect 83% of CRC cases in clinical studies [93].

Enhancing the potential value of liquid biopsy for CRC prognosis and prediction through the use of AI is a major frontier [124]. Recent studies shows that DL techniques and ML can significantly increase the analytical throughput and accuracy of TEP profiling. Next-generation RNA-seq was first used to profile TEP biomarkers in the design of novel platforms like ThromboSeq. This was then improved by adding techniques boosted by particle swarm optimization (PSO). By effectively searching the extensive platelet RNA-seq libraries to select the highly relevant RNA biomarker groups, these algorithms improve the diagnosis accuracy and refine the molecular signature for cancer categorization. Additionally, the advent of imPlatelet classifiers signaled a shift to DL applications, which transform TEP RNA-seq data into visual pictures. Even with small sample sets, the DL method enables precise cancer identification and risk assessment [89].

An innovative strategy for CRC diagnosis involves the isolation and detection of CTCs in blood samples [125]. The CellMax (CMx0) platform is an AI system that achieved 80% specificity and sensitivity in a cohort study of 47 patients [125]. Furthermore, according to a recent study based on ML approaches, AI applications could help analyze the content of particular blood protein biomarkers for the diagnosis of CRC with high accuracy (AUC = 0.86) [126].

Moreover, AI-based analysis of exosomal RNA and platelet-derived markers offers new perspectives on tumor development and patient classification [120]. Kandimalla et al. developed an innovative approach (EpiPanGI Dx), a cfDNA methylation test providing reliable diagnostic performance for GI malignancies [127]. The study examined DNA methylation patterns from 1,781 cancer samples and adjacent healthy tissues. They initially identified differentially methylated regions (DMRs) between GI cancers and normal tissues, as well as among various GI cancer types. All significant DMRs were incorporated and 67,832 tissue DMRs were prioritized. Then, tissue-specific DMRs were subsequently validated in 300 cfDNA samples. ML algorithms were employed to develop 3 different classes of DMR panels: cancer-type-specific biomarker panels, a pan-GI cancer detection panel, and a multi-cancer prediction panel for the majority of GI malignancies [127]. Moreover, the integration of AI-driven liquid biopsy data analysis with clinical data and imaging results can deepen disease comprehension allowing the implementation of individualized diagnostic and management approach [120].

AI-powered technologies also deliver enhanced accuracy and precision for early GI detection through fragmentomics, ctDNA mutation analysis, and methylation detection. Complementing methylation-based assays, fragmentomics analysis represents a cost-effective alternative for GI cancer screening. The DELFI approach utilizes AI to analyze genome-wide cfDNA fragmentation patterns. AI models trained on these fragment profiles can detect cancer with over 75% sensitivity in some cancers, by identifying abnormal chromatin packaging. In comparison to deep sequencing methods, the DELFI approach represents a cost-effective diagnostic tool as it uses low-coverage whole-genome sequencing, so it is promising for GI cancer screening programs [33, 115].

#### AI-powered liquid biopsy in early detection of hepatocellular carcinoma

HCC represents the predominant form of primary liver malignancy, constituting the sixth most frequently diagnosed cancer worldwide and the third major contributor to cancer mortality [1], with a rapidly increasing trend. The diagnosis and treatment of HCC present considerable difficulties owing to substantial inter- and intratumor heterogeneity [128]. Abdominal ultrasound is commonly used as diagnostic modality, but it has limited sensitivity in the detection of early-stage HCC due to cirrhotic liver echotexture and the requirement for sophisticated equipment [129]. Conventional tumor markers like AFP, AFP-L3, and DCP demonstrate insufficient sensitivity and specificity for early HCC identification [129]. While molecular characterization of tumors can reveal driver mutations offering insights into HCC’s genomic characteristics, yet single tissue samples from liver cancers may not adequately represent the tumor due to considerable intratumoral molecular diversity of HCC [130].

Liquid biopsy techniques offer numerous benefits including minimal invasiveness, rapid processing times, comprehensive mutational profiling, and the capability for repeated sampling that can deliver more precise representation of tumor dynamics [131]. By detecting genomic and molecular tumor characteristics, liquid biopsy has evolved as a promising minimally invasive strategy for HCC diagnosis, especially in early stages [132].

Integrating AI algorithms with the wealth of data obtained by liquid biopsy could provide a deeper comprehension of the underlying complex biological mechanisms of liver cancer, reveal new biomarkers, and facilitate development of more efficacious therapeutic modalities.

Analysis of cfDNA alterations implicated in HCC demonstrates considerable promise in HCC detection and clinical management, encompassing ctDNA mutations (CDKN2A and AXIN1 genes), DNA methylation changes (RASSF1A, SEPT9, KMT2C, and CCNA2 genes); and copy number variations (CDK6, EFGR, MYC, and BRAF genes) [133]. Nevertheless, this approach faces limitations due to minimal ctDNA quantities and challenges in distinguishing mutation signals from background interference [134].

Li et al. developed DL-based method called DISMIR model that can detect cancer in plasma cfDNA through the integration of methylation data and whole-genome bisulfite sequencing data [135]. DISMIR can define DMRs through a unique feature ‘switching region’. This method showed high accuracy in detection of HCC even with reduced sequencing depths [135]. Chen et al. developed an innovative integrated methodology for the diagnosis of HCC through the analysis of cfDNA. This method, named as 5-Hydroxymethylcytosine/motIf/Fragmentation/nucleosome footprint (HIFI), showed higher sensitivity and specificity performance than the traditional TM AFP [136].

Beyond genomics and epigenetics, exploration of key molecules in tumor metabolic reprogramming offers a new perspective for HCC liquid biopsy. For example, adenylosuccinate lyase (ADSL), a crucial enzyme in purine biosynthesis and cellular energy metabolism, has been reported to be dysregulated in multiple cancers (including HCC); it serves not only as an independent prognostic biomarker but is also closely linked to modulation of the tumor immune microenvironment [137]. Incorporating expression or mutational information for such metabolism-related genes into AI-based multi-omics models may further improve the sensitivity and specificity of early HCC detection. Likewise, glycerol-3-phosphate dehydrogenase 2 (GPD2), a glycolysis-related gene, has been shown to be an important prognostic biomarker in cholangiocarcinoma and to participate in tumor–immune interactions [138]. This highlights the significant potential of developing tumor type–specific AI recognition models tailored to the metabolic features of different hepatobiliary malignancies.

In addition, Yap et al. employed ML for the analysis of exosomal expression profiles from 118 patients with HCC and 112 healthy controls. A 9-exosomal RNA signature was identified from the examination of 114,602 exosomal RNAs that could predict HCC [139, 140]. Advanced AI algorithms can also process complex biomedical data enabling identification of liver metastasis arising from GI malignancies at early stage [136]. Advanced algorithms like CNNs and NLP could improve diagnostic accuracy through the analysis of complex genetic and molecular information, overcoming the limitations of traditional detection modalities like tissue biopsies and imaging [140].

Future integration of AI in liquid biopsy will advance clinical utilization of liquid biopsy for early HCC detection and management by addressing challenges related to low ctDNA quantities, and difficulties in distinguishing mutant signals from background interference [131].

### Role of AI-powered liquid biopsy in prognosis prediction and monitoring minimal residual disease

The convergence of liquid biopsy with ML is redefining the landscape of prognostication and disease surveillance [118]. The prognostic value of liquid biopsy lies in its sensitivity to subtle biomarker fluctuations that can forecast long-term outcomes. For instance, the detection of ctDNA has been shown to independently predict poorer survival, regardless of conventional clinical parameters [141].

One of the most clinically significant applications of this methodology is MRD monitoring, the small population of malignant cells that may persist after treatment and remain undetectable by standard imaging modalities [142, 143]. In CRC, ctDNA assays have revealed recurrences 6–9 months ahead of imaging, enabling clinicians to adapt treatment strategies or enroll patients in clinical trials earlier, thereby improving outcomes [144].

DL models like CNNs and RNNs could be used to identify cancer metastasis by examining cfDNA, miRNAs, CTCs, and protein markers [145]. An integrated approach combing DL frameworks with CNNs, and transformer-based DL architectures outperformed conventional biomarker-based assays in detecting metastatic cancer in blood samples with > 90% sensitivity and > 95% specificity [57]. The FDA approved CellSearch^®^ platform analyze CTCs in whole-blood specimens from patients diagnosed with metastatic CRC, breast, and prostate cancers, and showed promise as an important prognostic indicator of metastasis [118]. Integration of tumor-associated mutations with methylation or fragmentomics in plasma-only assays have reported one to one-and-a-half years between molecular relapse and clinical detection, with ctDNA negativity strongly associated with durable remission [146].

#### AI-powered liquid biopsy in breast cancer prognosis prediction

AI-based liquid biopsy could evaluate time-dependent variations in cfDNA mutations for early metastatic prediction in breast cancer using sophisticated models, such as an ensemble technique combining transformer-based DL architectures and CNNs [147, 148]. This approach showed excellent sensitivity (above 90%) and specificity (above 95%) in identifying metastatic signals from blood samples [57]. One study combined ctDNA and ML-predicted CTCs in metastatic breast cancer for endocrine resistance profiling, specifically in metastatic illness [149].

An example of AI-inspired solutions for prognostic modeling and predictive survival of breast cancer is the MammaPrint 70-gene expression signature which is a well-established tissue-based microarray/RNA-seq assays. It yields a binary risk score for breast cancer relapse providing comparison with molecular signatures in guiding patient management [140]. In addition, the IBM Watson for Oncology (WFO) system is an AI-driven clinical decision support, which uses ML and NLP to extract medical information. It was evaluated on 638 breast cancer cases to compare its treatment recommendations with those of a multidisciplinary tumor board, demonstrating 73% overall concordance in recommending therapeutic regimens [150].

Vora et al. used DL platform for the detection of rare CTCs and CTCs clusters in blood samples using flow cytometry which showed potential as biomarkers and predictors for metastatic growth [151]. In another study, Zhou et al. proposed ML model that could identify CTCs with very high efficiency, achieving 77.8% sensitivity and 97.56% specificity in predicting local recurrence or metastasis through the classification of CTCs in blood samples of breast cancer patients based on the epithelial–mesenchymal transition (EMT) state [152].

Automated microfluidic system integrating CTCs and HER2 expression offered high prognostic value in metastatic breast cancer [153]. Guo et al. designed a novel CNN method that could detect CTCs in peripheral blood samples to signal metastasis and predict prognosis with 97.2% sensitivity and 94% specificity based on immunofluorescence in situ hybridization images [154].

In a study by Gerratana et al., ctDNA characterization via NGS and CTC transcriptomic profiling using RNA sequencing were conducted in 1118 patients with luminal-like metastatic breast cancer. The study demonstrated that combining ctDNA and CTC analysis enabled enhanced comprehension of tumor mechanisms and therapeutic response in hormone receptor positive, HER2 negative metastatic breast cancer cases [155].

#### AI-powered liquid biopsy in lung cancer prognosis prediction

The integration of AI with ctDNA analysis presents a substantial utility in forecasting patient survival and estimating the risk of disease progression [101], facilitating the delivery of evidence-based, informed clinical decision-making [156, 157]. AI-driven analysis of liquid biopsy offers a dynamic means for monitoring tumor dynamics, which allows the precise detection of MRD and molecular relapse before clinical symptoms or radiological evidence emerges, thereby enabling more effective therapeutic interventions [106]. High-robust technologies such as digital polymerase chain reaction (dPCR), when coupled with AI, dramatically enhance the limit of detection for ctDNA, enabling the detection of uncommon sparse genetic variants related to tumor recurrence.

ML and DL algorithms are substantially improving prognostic accuracy in NSCLC by streamlining the analysis of comprehensive omics datasets while incorporating multiple clinical factors [158]. ML technologies can create prediction models that utilize genetic alteration data to anticipate disease trajectory and patient survival [101]. The consolidation of multi-layered omics information through AI frameworks can reveal how genetic variations influence disease advancement and patient prognosis [103].

A key application lies in the precise estimation of tumor mutational burden (TMB) from extensive whole-genome or targeted gene panel sequencing datasets [35]. TMB serves as a validated predictive marker for immunotherapy effectiveness, such as programmed-cell death (PD-1)/Programmed-cell death ligand-1 (PD-L1) inhibitors, with elevated levels typically associated with improved treatment responses [101]. Through AI-powered integration of TMB metrics with additional clinical factors, clinicians can formulate more personalized treatment regimens and generate prognostic information to guide long-term follow-up strategies [101].

#### AI-powered liquid biopsy in colorectal cancer prognosis prediction

The prognosis for CRC remains strongly associated with the stage at which it is detected, despite global endeavors to develop advanced treatment modalities. According to this perspective, liquid biopsy is a potential method that could assist medical professionals in disease screening, patient stratification for the most effective course of treatment, and routinely and minimally invasively monitoring tumor resistance mechanisms and treatment response [118]. Liquid biopsy provides prognostic and predictive insights by measuring ctDNA concentrations, which reflect tumor load and therapeutic response. Increased ctDNA levels are trustworthy indicators of the course of the disease since they have been connected to poorer clinical outcomes. Guardant360 is an AI-powered liquid biopsy test designed to direct treatment choices and tracking therapeutic outcomes through the analysis of ctDNA for the detection of somatic mutations in CRC [159, 160]. The inclusion of powerful DL architectures, such as CNNs and RNNs have improved the analysis of CTCs and ctDNA with enhanced sensitivity (above 90%) and specificity (above 95%) [2, 160].

Liquid biopsies are also essential for detecting MRD after chemotherapy or surgery through the detection of micrometastatic disease that might not be apparent on imaging, enabling early intervention and treatment regimen modification. Evidence suggests that individuals with detectable ctDNA following surgical intervention experience substantially elevated recurrence rates, warranting enhanced surveillance protocols and more intensive adjuvant treatment regimens [161]. The hybrid concept of CRC diagnoses, which combines molecular insights from liquid biopsy with Al-assisted endoscopic precision would transform risk assessment, early detection, and individualized therapeutic plans, ultimately improving patient outcomes.

Cancer prognosis continues to be the most prevalent clinical use of CTCs, with elevated CTC counts correlating with poorer patient outcomes. A prospective study examining pre-surgical stage I–IV CRC patients found that CTC detection rates, measured using CellSearch^®^ technology, were significantly higher in metastatic versus non-metastatic cases. For non-metastatic CRC patients, CTCs presence served as a robust independent prognostic marker, associated with reduced overall survival (OS) and progression-free survival (PFS) compared to CTC-negative individuals. When analyzing metastatic CRC patients specifically, CTC enumeration emerged as an independent predictor of both PFS and OS, with patients having ≥ 3 CTCs/7.5mL demonstrating shorter survival intervals than those with < 3 CTCs [118].

A promising, minimally invasive approach to effective management of CRC is the combination of AI and blood-derived molecular markers including cfDNA, peptides, and N-glycans. Although screening and early detection are the main uses of these AI-liquid biopsy techniques in current research, their value for prognosis prediction and individualized treatment decision-making is widely recognized [122]. Serum and plasma samples have been effectively analyzed using ML models, demonstrating the viability of using this sort of data for clinical classification. ML techniques, such as LR, SVM, RF, and LMT, have been utilized to examine N-glycan-based biomarkers and serum protein biomarkers based on mass spectrometry. Important peptides, like those from EGFR and leucine rich alpha-2-glycoprotein 1 (LRG1) have shown a significant degree of predictive power, with classification accuracy rates of up to 87% in differentiating advanced adenomas and CRC cancer from healthy individuals. This proves that a minimally intrusive blood biomarker technique is a viable substitute for traditional screening [122].

Employing the Union for International Cancer Control (UICC) II criteria, Hu and colleagues performed a modeling study that stratified 53 colon cancer patients into two cohorts—those experiencing post-surgical recurrence and those remaining disease-free—utilizing gene expression profiles. They suggested that the S-Kohonen neural network demonstrates superior effectiveness for colon cancer classification [162]. In a separate investigation, Xu et al. applied SVM analysis to identify differentially expressed genes capable of distinguishing high-risk recurrence patients. Notably, they identified a 15-gene signature with potential utility for predicting both prognosis and recurrence probability in colon cancer patients [162].

Emerging evidence highlights the critical role of non-coding RNAs in cancer detection and management. Identifying specific non-coding RNA expression alterations in colonic tissue and blood-derived samples could facilitate early CRC diagnosis, outcome prediction, and personalized treatment [163]. A Spanish study team used a strong predictive model to assess how a 6-miRNA signature in plasma samples distinguished healthy individuals and CRC patients using SVM classification model that showed 90% specificity and 85% sensitivity [164]. Clinicians can now forecast the prognosis of CRC patients using AI technology. Gründner et al. constructed predictive models using gene markers and various ML algorithms to assess disease-free survival (DFS), OS, radio-chemotherapy efficacy, and recurrence [165].

#### AI-powered liquid biopsy in liver cancer and hepatic metastases prognosis prediction

The combined application of AI with liquid biopsy methodologies has become a groundbreaking approach for preemptive identification of liver metastases originating from GI malignancies [140]. In the specific context of HCC, empirical studies have confirmed the clinical value of this integrated approach [129, 166]. For example, Lee et al. successfully combined a high-sensitivity cfDNA detection assay (utilizing PDA-SiO2 hybrids) with a specialized ML model for the detection and prognostic monitoring of HCC. Their approach integrated total plasma cfDNA levels with the expression of cfDNA derived from AFP (cfAFP-DNA) to generate a specific HCC score (cfDHCC). The cfAFP-DNA expression alone proved superior to traditional serum markers (such as AFP, AST, ALT) for discriminating HCC patients from control cohorts [166]. The ML-generated cfDHCC metric exhibited superior clinical performance, demonstrating enhanced precision in establishing patient UICC stages, identifying tumor multiplicity and microvascular infiltration (LVI), and approximating tumor dimensions. Additionally, the cfDHCC metric more accurately forecasted HCC relapse and survival compared to individual biomarkers evaluated in the investigation [129, 166]. Supporting research indicates that AI can analyze protein expression patterns to discover novel protein-based diagnostic and prognostic indicators in hepatic malignancies [167].

Although tissue sampling remains the definitive method for comprehensive mutation characterization in HCC, AI-enhanced liquid biopsy techniques utilizing CTCs and ctDNA are rapidly emerging as promising alternatives [168]. Analyzing ctDNA holds particular significance, as it comprehensively reflects the tumor’s overall genomic profile, attracting significant interest for determining clinical trajectories, especially in progressive HCC [19]. Mutational examination of ctDNA in advanced HCC cohorts has successfully detected alterations in key HCC-related driver oncogenes and tumor suppressor genes (such as TERT promoter, TP53, PTEN) and identified predictive mutation patterns associated with responsiveness to systemic treatments like tyrosine kinase inhibitors [169]. Postoperative persistence of ctDNA molecular signature was shown to be promising predictor of recurrence after liver resection for CRC patients with liver metastasis [170].

Despite ctDNA’s established clinical benefits, current challenges—including insufficient sensitivity for early-stage detection, absence of uniform protocols, and incomplete representation of tumor spatial diversity—highlight the critical need for multi-parameter strategies to improve its clinical application [171]. Consequently, while immensely promising for prognosis, AI-based liquid biopsy profiling presently serves as an adjunct tool for complicated scenarios rather than a substitute for conventional biopsy, awaiting rigorous confirmation through comprehensive large-scale prospective studies [155, 168].

The analysis of retrospective datasets using DL multi-omics models such as autoencoder/latent-space approaches, demonstrated prognostic performance through the stratification of TCGA and independent cohorts into survival subgroups. However, prospective interventional testing is still required to prove clinical utility [172]. For patients with HCC undergoing curative resection, postoperative ctDNA dynamics provide a molecular basis for a ‘watchful waiting’ strategy. Persistently negative ctDNA results can bolster clinician–patient confidence and help avoid overtreatment; the emergence of positivity indicates the need for intensified imaging assessments or early consideration of systemic therapy, effectively shifting the alert window from anatomical recurrence to molecular recurrence [173].

### Role of AI-powered liquid biopsy in informing treatment choices and advancing precision oncology

The combined use of liquid biopsy and ML is increasingly guiding therapeutic decision-making, central to the paradigm of precision oncology [174]. Through ctDNA assessment, tumor-distinctive genetic changes can be identified to direct personalized treatment strategies. The cobas^®^ EGFR Mutation Test v2 exemplifies this approach by detecting EGFR mutations in NSCLC [175].

Liquid biopsy also allows continuous surveillance of therapeutic response. Declines or rises in ctDNA levels can direct confirmatory imaging or prompt treatment modification, which is particularly essential in cases of acquired resistance, where tumors evolve mutations that reduce drug efficacy [176]. Detecting these resistance-associated mutations early allows clinicians to switch therapies at the most opportune time, potentially prolonging disease control and preserving quality of life [177].

The clinical validity of liquid biopsy for prognosis and therapy guidance is increasingly supported by prospective clinical trial data. SERENA-6 is a pivotal randomized study in breast cancer demonstrating that early detection of ESR1 mutations via liquid biopsy and subsequent switching to camizestrant markedly prolonged PFS and enhanced quality of life [178]. BESPOKE clinical trial aimed at assessing the value of ctDNA as a predictive and prognostic marker to direct postoperative treatment decisions in stage II/III CRC , where ctDNA-positive patients who received chemotherapy showed better recurrence-free survival compared to those untreated, thus personalizing treatment leading to better therapy outcomes [179]. In NSCLC, ctDNA tumor fraction detection has been related to poor outcome, establishing its utility as a clinically relevant prognostic and predictive biomarker [180]. Collectively, these trials illustrate the transition of liquid biopsy from an investigational technique to a clinically validated approach, underscoring its potential to reshape prognosis, treatment selection, and long-term patient management.

AI-driven liquid biopsy systems use ML to analyze a wide range of genomic, transcriptomic, and proteomic data from circulating biomarkers [181–183]. These advanced models can detect tiny changes in tumor size, reveal new resistance mechanisms, and forecast how effective a treatment might be before any symptoms appear. Because liquid biopsies can be performed repeatedly over the course of treatment, clinicians can monitor a patient’s progress continuously, making timely decisions about adjusting treatments or modifying regimens based on real-time data [184, 185].

The merging of AI and liquid biopsy is especially promising in tackling the problem of treatment resistance—an ongoing challenge in managing many kinds of cancers [186, 187]. Applying AI to analyze liquid biopsies has changed the scope of personalized medicine by enabling the evaluation of intricate multi-omics data that was previously too large and diverse for conventional techniques [188, 189]. ML can evaluate thousands of molecular features from circulating biomarkers, such as genetic mutations, epigenetic changes, protein levels, and metabolic markers. This comprehensive analysis helps reveal subtle biomarker patterns linked to how well a treatment works, the development of resistance, and patient outcomes [24, 190, 191].

In contrast to tissue biopsies, liquid biopsies can be done repeatedly during treatment to observe molecular changes as they happen. AI algorithms analyze these ongoing data streams to find trends and patterns that can accurately predict treatment success, allowing for proactive adjustments instead of reactive responses [192, 193]. People who cannot undergo repeated tissue biopsies due to tumor location or health issues, can benefit from sophisticated monitoring through simple blood tests, which is especially important for older patients and those with advanced disease [193, 194]. The future of response prediction involves multi-omics models integrating ctDNA mutations, methylation, EV-proteins, tumor transcriptomics, and metabolomics and are being advanced into prospective trials with embedded clinical decision support [195, 196].

Health economic analyses have demonstrated that liquid biopsy guided treatment strategies can reduce overall healthcare costs while improving patient outcomes and quality of life through individualized treatment and continuous monitoring. The ability to identify treatment resistance can prevent exposure to ineffective treatments and their related costs and toxicities [197]. These modern platforms will allow ongoing assessment of circulating biomarkers and integration with physiological parameters to provide comprehensive health assessments. The potential for these coeducational systems to transform cancer care through continuous, personalized monitoring constitutes an important development in precision oncology, promising to revolutionize how we monitor and manage cancer patients in the precision medicine era [198, 199].

#### AI-powered liquid biopsy in predicting treatment response in breast cancer

AI algorithms have demonstrated extraordinary capability in detecting and monitoring key driver mutations during treatment, including PIK3CA, ESR1, and ERBB2 alterations, through sophisticated ctDNA analysis platforms, identifying emerging resistance mechanisms and predicting treatment failure with remarkable accuracy [200, 201].

ML techniques have proven efficacy in analyzing the complex relationships between ctDNA kinetics and response to therapy in hormone receptor positive breast cancer patients treated with endocrine therapy [202]. AI powered platforms can model the intricate dynamics of ctDNA and appearance of resistance mutations including ESR1 alterations, that are related to acquired resistance to aromatase inhibitors and selective estrogen receptor modulators. These analyses provide clinicians with assessments of treatment efficacy weeks before radiological evidence of progression becomes evident [187]. The integration of AI with circulating tumor cell analysis has revolutionized the monitoring of breast cancer patients, particularly those with metastatic disease [203].

ML algorithms can analyze CTCs number, phenotypical characteristics, and molecular profiles to provide broad assessments of response to therapy and disease progression. Monitoring CTC dynamics during treatment has shown superior prognostic accuracy compared to traditional tumor markers such as CA15-3 and CEA, particularly in the initial phases of treatment when other biomarkers may remain stable [204]. AI has also enhanced the analysis of EVs from breast cancer patients, enabling the detection of treatment response and resistance patterns through analysis of exosomal cargo [89]. AI algorithms can decode complex molecular signatures in circulating exosomes, including miRNA profiles, lncRNAs, and protein markers that reflect tumor biology and therapeutic response. These EVs based analyses have shown promise in TNBC, where traditional biomarkers are often inadequate for treatment monitoring [205].

The integration of DL platforms has enabled the deep analysis of multi-omics data to simultaneously analyze genomic, transcriptomic, and proteomic information from liquid biopsies to generate prognostic models with superior accuracy compared to single modality approaches [206]. This has particular importance in breast cancer, where tumor heterogeneity can limit the prognostic value of individual biomarkers [207]. Dong and colleagues used a multi-omic framework linking genomics, transcriptomics, and serial ctDNA to show that MRD signatures in the neoadjuvant setting outperformed imaging alone for predicting recurrence, enabling stratification of responders and non-responders to support escalation or switch strategies guided by ctDNA status [208].

Recent advances include the development of platforms specifically designed for monitoring patients receiving CDK4/6 inhibitors in hormone receptor positive breast cancer [186, 209], that can predict treatment response and identify mechanisms of resistance through analysis of ctDNA mutations and cell cycle related protein markers, which has significant implications for optimizing treatment duration [181, 210]. The monitoring of MRD following breast cancer treatment represents another application [182, 183]. Following operative resection and adjuvant therapy, AI enhanced platforms can detect trace amounts of ctDNA that could indicate the existence of micrometastatic disease and guide decisions regarding adjuvant therapy and surveillance strategies [184, 185].

ML approaches have also proven important in analyzing the immune microenvironment of breast cancer particularly in patients receiving immunotherapy through the analysis of circulating immune markers, including T cell receptor diversity, cytokine profiles, and immune checkpoint protein levels, to provide insights into immune system activation and tumor immune evasion mechanisms [211]. AI has enabled the development of prognostic models that can analyze ctDNA in breast cancer patients receiving neoadjuvant therapy to predict response and guide surgical intervention [190]. Several studies have shown that AI powered liquid biopsy approaches can guide treatment decisions with high precision compared to tissue-based analyses and providing real time monitoring throughout the treatment course [24]. Given the high cost of targeted therapies and the importance of precise treatment monitoring, the economic benefits of AI integrated liquid biopsy in breast cancer are substantial [187].

#### AI-powered liquid biopsy in predicting treatment response lung cancer

The combination of AI and liquid biopsy analysis has changed the approach of management of NSCLC patients, particularly those on targeted therapies. Through ctDNA analysis, these AI systems are capable of detecting and tracking gene mutations, including important resistance mutations like T790M mutation in EGFR [212]. They can detect signs of resistance before clinical symptoms become evident, often weeks or months earlier, enabling timely intervention and improved prognosis.

ML is especially helpful in monitoring the reduction of ctDNA levels during treatment, serving as a reliable marker for treatment response [213, 214]. AI-powered tools can analyze the complex relationships between ctDNA quantities, tumor size, and response to therapy, giving healthcare providers clear, quantifiable insights. These methods have been shown to predict treatment success more accurately than traditional imaging, especially early on before changes can be detected by imaging techniques.

AI also plays a key role in forecasting how patients will respond to immunotherapy [215]. Biomarkers like PD-L1 expression alone have low predictive power because tumors are so diverse, and their expression can change. With AI-enhanced liquid biopsy techniques, multiple circulating markers—such as ctDNA, CTCs, and immune-related proteins—can be analyzed together, offering a more detailed picture of immune activity and how tumors evade immune responses [216, 217].

Using DL to analyze combined molecular data from liquid biopsies has further improved lung cancer stratification and prediction of response to therapy [218]. These advanced algorithms bring together genetic, transcriptomic, and proteomic information to create detailed molecular portraits of tumors, allowing for more personalized therapy [219]. ML tools can also analyze CTC counts, cell characteristics, and molecular profiles to gauge treatment effectiveness and identify resistance, enabling more personalized treatment plans [220, 221].

Utilizing AI-based tools to analyze EVs from lung cancer patients is an exciting new development in the field [198]. These advanced systems can interpret complex molecular details in circulating EVs, exosomal RNA and proteins, which is very promising in monitoring response to therapy particularly during immunotherapy where traditional markers have limited sensitivity [222].

Recent clinical research has emphasized how AI-driven liquid biopsy methods are proving useful in managing lung cancer. Studies have shown that these tools can help guide treatment choices with accuracy that matches or even surpasses traditional tissue tests [223]. Because liquid biopsies provide real-time information, clinicians can adjust treatment based on changing molecular profiles, rather than waiting for scans or symptoms to show progress or setbacks [224].

These AI-powered liquid biopsy techniques also have important cost benefits, potentially reducing the need for repeated tissue biopsies, scans, and hospital stays [225]. Cost analyses suggest that using liquid biopsies to guide treatment can improve patient outcomes while lowering overall healthcare expenses, especially in advanced lung cancer cases where options are limited and resistance to therapies is common [226, 227]. Looking ahead, future improvements in AI-based liquid biopsies for lung cancer include combining data from real-world evidence and electronic health records to improve prediction accuracy [228]. These integrated platforms will blend molecular data from blood tests with clinical, demographic, and treatment history information, leading to even more precise forecasts of treatment response [2].

#### AI-powered liquid biopsy in predicting treatment response in colorectal cancer

The molecular complexity of CRC owing to the heterogeneity of driver mutations, MSI status as well as various resistance mechanisms foments an AI-enhanced analytics approach fitting for integrating multiple biomarker modalities for a comprehensive treatment response assessment [229].

By leveraging AI to study circulating biomarkers from CRC patients, complex predictive models on treatment outcomes were designed with very high prediction accuracy. ML methods have been especially successful at examining the complex mutation patterns of ctDNA that define CRC tumors including APC, KRAS, PIK3CA, and TP53 alterations [230]. Such AI-based platforms could detect the emergence of genetic variants that could be associated with treatment resistance and hence may be able to predict treatment failure prior to clinical progression [186, 209].

Integrating liquid biopsy and AI algorithms has transformed the practice of monitoring for CRC patients on targeted regimens. Anti-EGFR therapies are effective only in CRC patients having RAS wild-type tumors and their continued clinical benefit relies on monitoring the appearance of resistance mutations during treatment [230]. AI-driven liquid biopsy platforms can detect these resistance mutations with high precision, thereby enabling the adjustment of therapeutic strategies at an early time point improving patient outcomes [193].

ML-based tools have also been of significant value in the analysis of CTCs in patients diagnosed with CRC, especially for the evaluation of treatment response and prognosis prediction [24, 190]. ML CTC-based approaches can provide a holistic assessment of treatment response based on CTC profiling in terms of enumeration, morphology and biology. The ability to track CTC dynamics through therapy has shown great promise in predicting OS and informing clinical decisions [191, 192].

Moreover, an AI method used for the analysis of methylation pattern from circulating DNA has been recognized as a significant development in CRC liquid biopsy [181, 210]. Epigenetic changes play a crucial role in the CRC pathogenesis and treatment response, and AI – based platforms can interpret complex methylation signatures that are linked to response to treatment. These methods have exhibited promising ability in monitoring subjects receiving immunotherapy, as methylation status can affect treatment response [182, 183].

Contemporary developments in AI mediated liquid biopsy for CRC includes the establishment of an exosome-based analysis platform to predict treatment response [184]. Such innovative systems analyze the molecular contents of circulating EVs, such as miRNAs, lncRNAs, and proteins to provide comprehensive profiles of tumor biology and treatment response. Using ML, algorithms can be generated to make predictions based on these multi-dimensional datasets that can guide therapeutic choices [185].

Integration of AI to the liquid biopsy methods has greatly enriched capabilities for monitoring MRD in CRC patients who underwent surgical operations [231]. Patients with ctDNA detectable by AI-assisted platforms could receive early treatment for micrometastatic disease and potentially benefit from the improved outcomes. These strategies have shown potential in patients diagnosed with stage II–III CRC, where MRD can guide adjuvant therapy [232, 233]. Furthermore, AI has proved highly useful for examining alterations in the level of circulating biomarkers during CRC treatment providing insights into treatment response and resistance development [234].

ML algorithms can model the complicated relationships between biomarker levels, tumor burden, and treatment efficacy, enabling further precise prediction of treatment outcomes. These analyses have shown superior prognostic accuracy compared to single time point assessments, highlighting the importance of longitudinal monitoring in precision medicine approaches [118, 235]. The application of learning networks has enabled the integration of imaging and molecular data to provide inclusive treatment response assessments [118, 235]. These platforms can analyze radiological images alongside liquid biopsy data to provide further correct assessments of treatment efficacy and disease progression. The ability to combine multiple data modalities through AI enhanced analysis represents a significant advancement in CRC management [236].

Validation studies have demonstrated the utility of AI powered liquid biopsy approaches can guide treatment decisions with accuracy compared to tissue-based analyses while providing real time monitoring [187, 237]. The non-invasive nature of liquid biopsy permits frequent assessments throughout treatment course, allowing for dynamic optimization of therapeutic strategies based on evolving molecular profiles [118, 238], reducing the need for invasive procedures, imaging studies, and hospitalizations while improving treatment outcomes [199, 218]. In addition, liquid biopsy guided treatment strategies can reduce overall healthcare costs through accurate response prediction while improving patient quality of life and survival outcomes [198, 214].

#### AI-powered liquid biopsy in predicting treatment response in hepatocellular carcinoma

New advances in AI enabled the development of sophisticated platforms that can monitor treatment response and guide decision making in liver cancer patients with unprecedented accuracy [131]. The convergence of nanotechnology and AI in liquid biopsy applications has shown particular promise in liver cancer, where traditional biomarkers have often proven incapable for treatment monitoring [239]. AI enhanced platforms can analyze complex patterns of biomarkers which portrays the unique biology of liver tumors, distinguishing between tumor-related and liver disease related biomarker changes, providing more accurate assessments of treatment efficacy [240, 241]. While AFP alone has limited utility as a treatment response biomarker owing to its variability and lack of specificity, AI powered platforms can integrate AFP levels with other circulating markers to generate predictive models with significantly improved accuracy to provide comprehensive treatment response assessments in HCC patients [242, 243]. These integrated approaches have shown promise in monitoring patients receiving immunotherapy and targeted therapy regimens [244].

It was also shown that combining circulating markers, like AFP and des-gamma-carboxy prothrombin (DCP), with transcriptomic, proteomic and spatial immune profiling (for example, single-cell RNA-seq and spatial transcriptomics that map immune cell subsets and checkpoint ligand expression) can increase diagnostic accuracy and better capture immune evasion phenotypes that drive treatment resistance [245].

The high background of noncellular DNA from liver cells, particularly in patients with underlying liver disease, can complicate ctDNA analysis. However, AI algorithms can distinguish between tumor derived and liver derived DNA fragments through sophisticated pattern recognition approaches that analyze sequence characteristics, methylation patterns, and fragmentation profiles [112, 186]. AI enhanced liquid biopsy platforms have shown remarkable success in monitoring liver cancer patients receiving systemic treatments, such as immune checkpoint inhibitors, Sorafenib and Lenvatinib [130, 246]. These sophisticated systems can predict treatment response within weeks of therapy initiation, enabling primitive identification of non-responders and timely modification of treatment regimens.

Monitoring treatment effectiveness in real time has particular importance in liver cancer, where treatment options are limited and early identification of resistance is crucial for improving patient outcome [140, 143]. The integration of AI with EVs has evolved as a clinically potential approach in monitoring liver cancer [209, 247]. Hepatocytes and liver cancerous cells release abundant EVs to the circulation, and these vesicles carry molecular cargo that reflects the tumor microenvironment and treatment response status [181, 182].

While CTCs are comparatively rare in liver cancer patients compared to different tumor types, AI enhanced detection and analysis platforms have improved the sensitivity and clinical utility of CTC based assessments through the analysis of CTC quantity, morphology, and molecular profiles to assess treatment response [248, 249]. The application of DL platforms can simultaneously analyze multiple biomarkers like ctDNA, EVs, CTCs, and protein biomarkers to generate treatment response predictive models with superior accuracy compared to individual biomarker approaches [187, 189]. AI has also proven eminent in investigating the metabolomic profiles of plasma and serum from liver cancer patients, providing insights about tumor metabolic pathways [250]. The monitoring of residual disease following liver cancer treatment represents another eminent application of AI powered liquid biopsy technologies. Following operative resection or liver transplantation, AI can detect trace amounts of circulating biomarkers that may indicate the presence of relapse or residual disease enabling timely intervention and enhanced prognosis [251].

The economic implications of AI powered liquid biopsy in liver cancer are particularly significant given the high cost of systemic therapies and the importance of early response assessment [252]. Cost effectiveness analyses have shown that liquid biopsy guided treatment strategies can reduce overall healthcare costs while improving patient outcomes. Early identification of non-responders in the initial phases of treatment course can prevent unnecessary exposure to ineffective therapies [252]. Up-coming directions in AI powered liquid biopsy for liver cancer include the development of integrated platforms that combine molecular analysis with AI enhanced imaging interpretation [253, 254]. The integration of molecular and imaging data through sophisticated AI algorithms represents the next frontier in precision medicine for liver cancer patients.

## Navigating the challenges and limitations

The previous sections demonstrated technical achievements and promising clinical applications of AI-driven liquid biopsy across multiple cancer types. However, the path to widespread clinical adoption remains fraught with substantial hurdles that must be addressed. Translating AI algorithms to diverse healthcare systems and varied clinical workflows requires overcoming technical, biological, clinical and ethical barriers **(**Table 3**).** Understanding these challenges is essential for implementation to enhance precision oncology.

**Table 3:** Navigating the Challenges and Limitations of liquid biopsy

Challenge Category	Specific Challenge	Impact on Prognosis and Clinical Utility
**Technical**	Low analyte concentration and shedding	Increased risk of false negatives, especially in early-stage tumors or “non-shedding” cancers. Leads to inconclusive results that require follow-up with a tissue biopsy [255, 256].
	Need for deep sequencing	Increases the cost and technical complexity of assays, which can limit the genomic scope of liquid biopsy panels [257].
	Pre-analytical errors	Degradation of analytes can lead to unreliable and uninterpretable results, undermining the accuracy of the test [258].
**Biological**	Clonal hematopoiesis (CH)	Benign mutations from white blood cells can contaminate the sample, leading to false positives and complicating the interpretation of a positive result, particularly for common mutations like *TP53* and *KRAS* [259, 260]
**Clinical/Ethical**	Lack of standardized protocols	Inconsistency in results across different laboratories, hindering widespread clinical validation and implementation [261, 262]
	Over-interpretation of results	The risk of clinicians making aggressive treatment decisions based on an uncertain positive result (e.g., ctDNA present but no tumor on imaging) or a potentially misleading negative result [263, 264]

### Technical hurdles: from sample to analysis

**Fig. 5:**
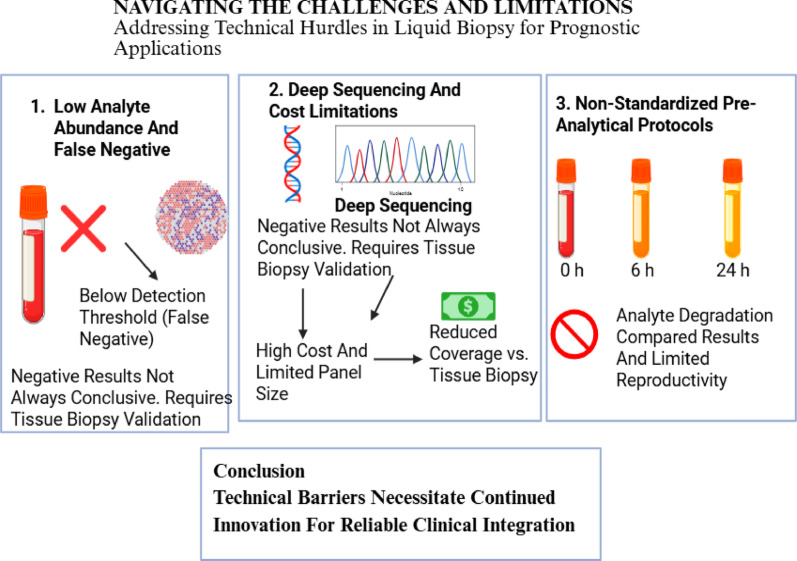
Technical hurdles in liquid biopsy for prognostic applications. Created in BioRender. Galal, S. (2026) https://BioRender.com/m8hfnhs

While liquid biopsy holds remarkable promise for prognostic applications, its routine integration into clinical practice is hindered by several technical barriers (Fig. 5). A central challenge is the limited availability of tumor-derived analytes in blood, particularly in patients with early-stage disease [3]. This scarcity increases the likelihood of false-negative results, as clinically relevant genetic alterations may be present at levels below the detection threshold of current assays. As a result, a negative liquid biopsy cannot always be considered conclusive and often necessitates validation through tissue biopsy [265].

Another obstacle lies in the requirement for deep sequencing to achieve sufficient sensitivity and accuracy. Compared to tissue biopsies, liquid biopsy assays must interrogate genomic regions at much greater depth to reliably capture rare variants [57]. This demand not only escalates costs but also constrains the practical size and breadth of genomic panels, thereby limiting their coverage relative to tissue-based analyses [266].

NGS technologies have revolutionized liquid biopsy through their capacity to generate comprehensive genomic, epigenomic, and transcriptomic profiles from minimal sample volumes [267]. Modern NGS methods have developed beyond basic mutation detection to the creation of high-dimensional, multiparametric datasets that include fragmentomic profiles, methylation status, and genomic variations. AI architectures with multimodal integration capabilities are required to analyze this complexity [268]. In particular, by considering sequencing pileups as image-tensors, CNNs are being used for advanced error correction, successfully lowering false positives in low-frequency variant calling. Additionally, by using Self-Attention processes to assign weights to the most clinically important characteristics, Transformer-based models enable the merging of different data sources, such as linking cfDNA fragment length with epigenetic silencing. To achieve the sensitivity needed for early-stage cancer detection, rule-based filters must give way to AI-driven multiparametric analysis and providing the robust frameworks needed to translate liquid biopsy findings into actionable, evidence-based care pathways [269, 270].

Furthermore, the absence of **standardized pre-analytical protocols** represents a critical bottleneck. Variables such as the interval between blood collection and plasma separation can markedly influence assay reliability, with delays leading to analyte degradation and compromised results [271]. Without harmonized protocols across laboratories, reproducibility and clinical confidence in liquid biopsy assays remain restricted.

### The confounding factor of clonal hematopoiesis

One of the key biological obstacles in liquid biopsy interpretation is the interference from **CH**—the process of emergence of somatic mutations in normal hematopoietic cells, leading them to release DNA fragments into circulation [272]. This phenomenon can introduce non-tumor derived mutations into liquid biopsy samples, complicating the distinction between malignant and benign genetic alterations.

Although detection of a clinically relevant mutation in a liquid biopsy is generally regarded as highly reliable, CH undermines the central assumption that all ctDNA originates from malignant tissue [273]. Well-known oncogenic mutations, including alterations in *TP53* or *KRAS*, may instead stem from benign hematopoietic clones rather than tumor cells, creating the risk of misattribution [273]. Without careful discrimination, such results could mislead clinicians, yielding inaccurate prognostic interpretations or inappropriate therapeutic strategies. Consequently, laboratories must employ specialized workflows and analytical rigor to distinguish tumor-derived DNA from CH-related variants, highlighting the necessity for cautious and contextualized clinical interpretation [273].

To address this challenge, several algorithms now compare cfDNA mutation frequencies with matched leukocyte genomic sequencing results, or leverage features such as fragment size and end-motif sequences to distinguish tumor- from CH-derived mutations, thereby reducing false positives [260, 274].

### Overcoming clinical and ethical barriers

**Fig. 6:**
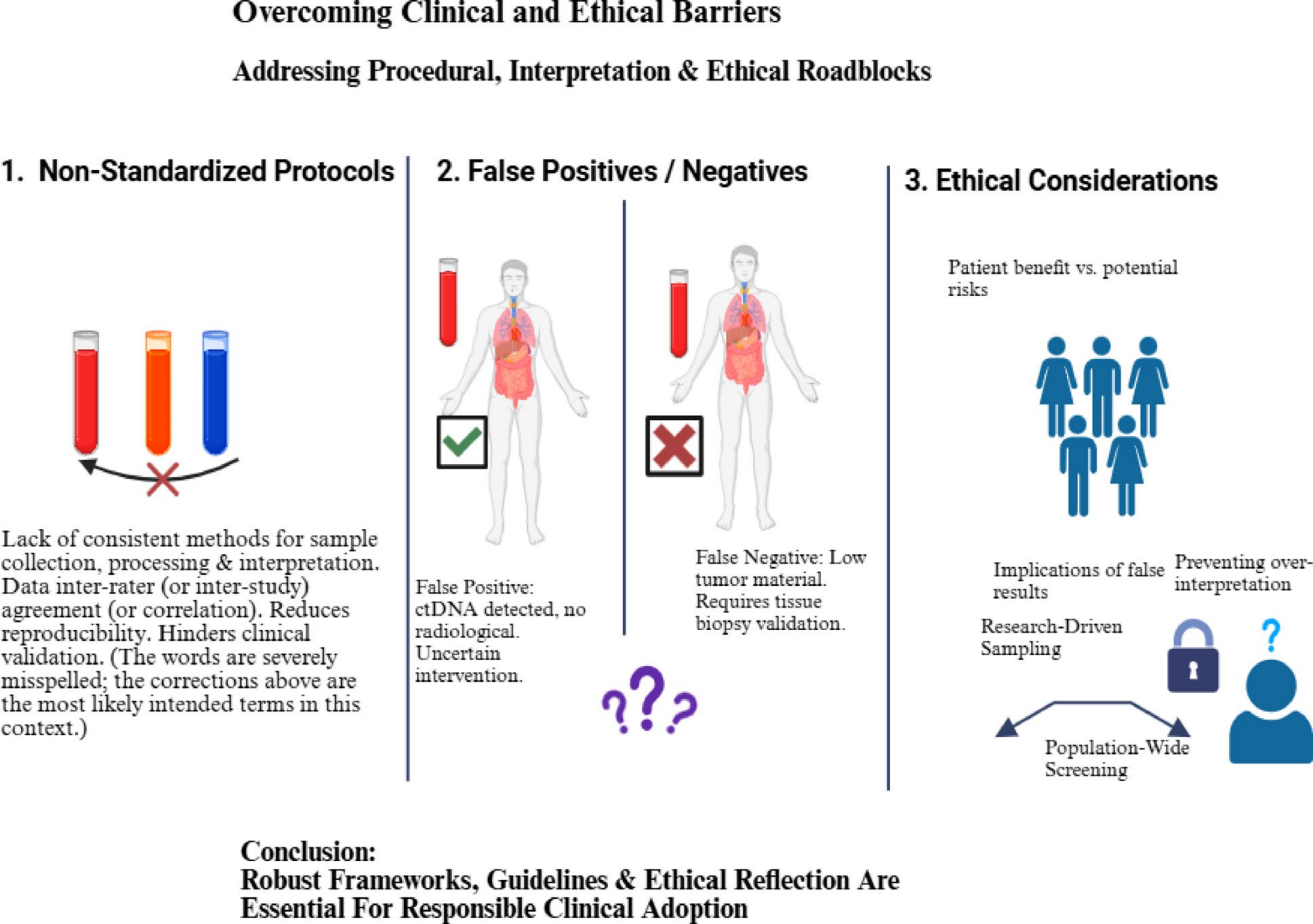
Overcoming Clinical and Ethical Barriers. Created in BioRender. Galal, S. (2026) https://BioRender.com/d27hdtp

In addition to technical and biological hurdles, the broader clinical adoption of liquid biopsy for prognostic use is constrained by both procedural and ethical barriers (Fig. 6). A major limitation is the **absence of standardized protocols** pertaining to sample collection, sample processing, and data interpretation, which reduces reproducibility between laboratories and impedes clinical validation [275, 276]. Without harmonized methodologies, integration into routine oncology practice remains premature.

The risks of **false positives and false negatives** further complicate clinical application. For instance, a patient may test positive for ctDNA despite the lack of radiological evidence of cancer. Such scenarios raise uncertainty about whether immediate, aggressive intervention is warranted, underscoring the absence of clear clinical guidelines for interpreting discordant results [277, 278]. This gap highlights the urgent need for robust frameworks that translate liquid biopsy findings into actionable, evidence-based care pathways.

Ethical considerations also warrant careful reflection. Although liquid biopsies are far less invasive than traditional procedures, their application in contexts such as **research-driven sampling** or **population-wide screening** introduces complex issues. These include balancing patient benefit against potential risks, managing the implications of false results, and preventing over-interpretation of ambiguous findings [3]. Addressing these ethical obstacles crucial to guarantee proper deployment of liquid biopsy in both clinical and public health settings.

Beyond improving diagnostic precision, AI-driven liquid biopsy has a profound impact on patient care in the real world. Clinicians can transition from static snapshots to dynamic monitoring by utilizing RNN-based longitudinal analysis, which can detect molecular recurrence months ahead of conventional imaging. Additionally, AI’s capacity to combine multimodal data, for example, connecting proteomic expression and genetic variants of unknown significanceV, offers a workable way to resolve diagnostic uncertainty at the bedside. These benefits enable a precision oncology framework that optimizes therapeutic windows and lessens the clinical burden of false results by allowing treatment to be modified in real-time based on the tumor’s changing genetic landscape [279, 280].

Nevertheless, the “black-box” nature of AI is a major challenge in medicine because AI models produce unexplainable outputs, and clinicians need to understand how predictions and decisions are made [281, 282]. Explainable AI (XAI) frameworks, like SHapley Additive exPlanations (SHAP) and Local Interpretable Model-agnostic Explanations (LIME), can provide partial interpretability by explaining features attributions, building trust and enabling clinical validation [283, 284]. In the context of cfDNA-based liquid biopsy, SHAP quantifies the contribution of each methylation site to the model’s output, assigning scores that reveal which methylation features drive clinical predictions enabling researchers to identify specific genomic regions and epigenetic alterations responsible for cancer classification decisions. Similarly, LIME generates interpretable models that explain individual predictions by approximating complex model behavior through simpler surrogate models, enabling clinicians understanding of the rationale behind specific patient classifications [285]. Nevertheless, improved interpretability of XAI is essential in trustworthy clinical adoption AI-driven liquid biopsy data [284].

## Future trajectory and strategic recommendations

Despite the broad prospects of AI-enabled liquid biopsy, clinical translation still faces several bottlenecks, including insufficient sensitivity in early disease, tumor heterogeneity, and a lack of effective therapeutic targets. Nevertheless, promising future directions and strategic recommendations can substantially aid in advancing liquid biopsy and transforming the integration of AI platforms to become a routine component of precision cancer care accessible across diverse healthcare settings.

### Multi-omics integration: beyond a single biomarker

The future of liquid biopsy and ML for prognosis lies in moving beyond the analysis of a single biomarker. A single biomarker is often an unreliable prognostic predictor due to the inherent variability of disease expression across individuals. The complexity of tumor biology and its dynamic evolution require a more comprehensive approach [66].

This future is centered on the integrative analysis of multi-omics data. Through the convergence of data from different biological layers—such as genomics (ctDNA), epigenetics (methylation patterns), transcriptomics (RNA), and proteomics—ML models can build a more holistic and robust view of a patient’s disease. This multimodal approach has the potential to reveal system-level insights that could not be gleaned from a single data type alone, and it is anticipated to outperform single-modality analysis. This integrative strategy is a critical step toward building more accurate and reliable prognostic models that can fully support the goals of personalized oncology [36], as well as direct drug discovery [286].

However, a core challenge in multi-omics liquid biopsy approaches is merging heterogenous data types as genomics, methylomics, transcriptomics, and proteomics that differ in scale, dynamic range, resolution, sparsity, and dimensionality [287]. AI frameworks address these discrepancies using explicit algorithmic weight allocation at the feature, modality, and sample levels [34]. Ensemble learning methods combine outputs from multiple models trained on different omics layers. Three primary fusion strategies have emerged: early fusion (feature-level integration), late fusion (decision-level integration), and intermediate fusion (mode-level) with attention mechanisms [288]. In early fusion, features from multiple omics layers are directly integrated into unified input vectors, allowing models to capture complex cross-modal interactions but requires availability of all data types simultaneously. Late fusion instead trains independent models on each omics layer and integrates their outputs through weighted voting or stacking. Intermediate fusion utilizes transformer architectures and cross-modal attention mechanisms to guide models to focus on interconnected features across omics layers by allocating weights based on sample-specific patterns and biological context. Other architectures include graph neural networks that models molecular relationships as graph structures, and multi-modal variational autoencoders that map heterogeneous omics data into unified embedding spaces [289]. Collectively, these adaptive weighting strategies have been shown to improve the performance and interpretability of multi-omics liquid biopsy for early cancer detection and treatment response monitoring compared with single-omics approaches.

### A new frontier: integrated diagnostics with imaging and AI platforms

The future of liquid biopsy is not as a standalone test but as an integrated component of a broader, multimodal diagnostic ecosystem. This new frontier involves the fusion of liquid biopsy technologies with advanced imaging devices like MRI and CT scans. Liquid biopsy offers dynamic, molecular-level insights, while imaging provides a macroscopic, anatomical context of the tumor. Integrating these data streams allows for enhanced diagnostic precision; for instance, a change detected in ctDNA levels could prompt a follow-up imaging scan to assess physical changes in the tumor, enabling more precise and timely adjustments to treatment plans [7].

This integration is being facilitated by AI-enabled platforms that can assimilate and analyze these large amounts of complex, multi-source data. These platforms can use algorithmic models to provide additional insight, recommend targeted therapies, forecast treatment effectiveness, and match patients to clinical trials. This synergy of data streams and computational power will revolutionize how clinicians make decisions, paving the way for a truly personalized and proactive cancer management approach [290].

### Overcoming multi-center validation performance discrepancies

The clinical implementation of AI-driven liquid biopsy platforms faces the challenge of the performance discrepancies observed across multi-center validation studies originating from multiple factors [245]. These include: patients’ population heterogeneity including differences in ethnicity, cancer epidemiology, age distribution, and comorbidities across; technical variability arising from sequencing platform differences, library preparation protocols, and bioinformatics analysis workflows; manufacturing batch variations including reagent chemistry, and laboratory-specific handling procedures; and calibration issues when disease prevalence differs between training and validation settings [291].

Strategies to improve robustness of AI models include training models on ethnically and geographically diverse multi-institutional cohorts, selecting biologically-driven features less susceptible to technical variability, implementing domain adaptation techniques that explicitly model site-specific variation, and employing federated learning approaches (FL) that enable collaborative model development without centralizing patient data [289].

### Recommendations for future research and clinical adoption

Understanding and exploiting the complete potential of liquid biopsy integrated with ML for cancer prognosis demands multiple strategic initiatives:

1. **Continued Clinical Validation**: The most pressing need is the conduction of large-scale, prospective, blinded, multi-center clinical trials to verify the clinical potential of these technologies across cancer types as well as stages. This will build the necessary body of evidence to move the technology from research to the clinical setting [7]. Future clinical trial designs should strive to actively integrate dynamic liquid biopsy monitoring with emerging therapeutic strategies, such as metabolic targeting and combination immunotherapy [292].
2. **Standardization of Protocols**: International consensus on pre-analytical and analytical protocols should be established. The development and widespread adoption of standardized protocols pertaining to sample collection, handling, and analysis are critical to ensure the consistency and reliability of results across different institutions and labs [293].
3. **Technological Advancement**: Continued investment in technological advancements is needed to enhance the sensitivity and specificity of assays, particularly for the detection of low-concentration analytes in early-stage cancers [24].
4. **Educational Initiatives**: Healthcare providers should be trained on interpreting and acting on ML-guided liquid biopsy results, particularly the difference between a reliable positive result and a potentially unreliable negative one [24].
5. **Ethical and Regulatory Guidelines**: Clear ethical and regulatory guidelines must be established to address the risks of over-interpretation, the use of mandatory biopsies in clinical trials, and the future potential for population-wide cancer screening [24].
6. **Decentralized Data Training via Federated Learning**: The main challenge in improving AI models for clinical use is the restricted access to large-scale, private genomic datasets owing to the strict application of data-protection laws. To get around the limitations of data “silos” and strict privacy rules, future research should use FL. Unlike traditional centralized methods, FL lets AI models train across many hospitals and institutions without needing to share raw patient data. Kaissis et al. explained that just the locally computed model updates (gradients) are shared, which provides a ‘privacy-by-design’ framework that safeguards sensitive information while still making it possible to develop strong, multi-institutional diagnostic tools [294]. FL is currently being recognized as a promising option to allow sharing big data across institutions in a way that adheres to the EU General Data Protection Regulation (GDPR). FL is increasingly combined with privacy-enhancing technologiessuch as Differential Privacy to prevent the identification of individual patient data, and Secure Multiparty Computation (SMPC), which enables encrypted computation [295].

## Conclusion and future perspectives

The successful integration of AI and liquid biopsy technologies represents a paradigm shift in oncology field, offering unprecedented opportunities to transform cancer care to more proactive and individualized approaches. The current review deeply addressed how ML algorithms could analyze complex, multi-dimensional data generated by liquid biopsies, enabling earlier detection of cancer, more accurate prognostication, patient stratification, real-time treatment surveillance, and more precise personalized therapeutic decisions. Nevertheless, harnessing the full capabilities of liquid biopsy and ML for cancer management requires coordinated endeavors across technical innovation, clinical validation, regulatory approval, to enable healthcare system implementation. By tackling these hurdles, the oncology field can confidently move toward a future where liquid biopsy, powered by ML, is a cornerstone of precision oncology, providing critical, real-time prognostic information that leads to better-informed decisions and improved patient outcomes.

## Data Availability

No datasets were generated or analysed during the current study.

## Abbreviations

AI: Artificial intelligence
ADSL: Adenylosuccinate lyase
ASCENDx: Acoustic Separation and Concentration of Exosomes and Nucleotide Detection
CA15-3: Carbohydrate antigen 15 − 3
CA19-9: Carbohydrate antigen 19 − 9
CEA: Carcinoembryonic antigen
cfAFP-DNA: cfDNA derived from alpha-fetoprotein
CH: Clonal hematopoiesis
CLiP: Lung Cancer Likelihood in Plasma
CMx0: CellMax
CNNs: Convolutional Neural Networks
Cox-PH: Cox proportional hazards
CRC: Colorectal Cancer
CTCs: Circulating tumor cells
ctDNA: Circulating tumor DNA
dPCR: Digital polymerase chain reaction
DL: Deep learning
DELFI: DNA evaluation of fragments for early interception
DMR: Differentially methylated regions
DTs: Decision Trees
DFS: Disease-free survival
EGFR: Epidermal growth factor receptor
EMT: Epithelial–mesenchymal transition
EVs: Extracellular vesicles
FL: Federated learning
FDA: Food and Drug Administration
GI: Gastrointestinal
GDPR: General Data Protection Regulation
GPD2: Glycerol-3-phosphate dehydrogenase 2
HCC: Hepatocellular carcinoma
HIFI: 5-Hydroxymethylcytosine/motIf/Fragmentation/nucleosome footprint
KNN: K-Nearest Neighbor
LncRNA: Long non-coding RNA
LDCT: Low-dose computed tomography
LR: Logistic regression
LIME: Local Interpretable Model-agnostic Explanations
LRG1: Leucine rich alpha-2-glycoprotein 1
MCED: Multi-cancer early detection
ML: Machine learning
mRNA: Messenger RNA
miRNA: MicroRNA
MRD: Minimal residual disease
NLP: Natural language processing
NSCLC: Non-small cell lung cancer
NNs: Fully Connected Neural Networks
oncRNAs: Orphan non-coding RNAs
OS: Overall survival
PD: Programmed-cell death
PD-L1: Programmed-cell death ligand 1
PFS: Progression-free survival
PSO: Particle swarm optimization
RFs: Random Forests
SMPC: Secure Multiparty Computation
SHAP: SHapley Additive exPlanations
SPOT-MAS: Screening for the Presence of Tumor by Methylation and Size
SVMs: Support Vector Machines
TNBC: Triple negative breast cancer
TEPs: Tumor educated platelets
TIRF: Total Internal Reflection Fluorescence
TNBC: Triple negative breast cancer
UICC: Union for International Cancer Control
WFO: Watson for Oncology
XAI: Explainable artificial intelligence
